# Cryptochrome 1 promotes photomorphogenesis in Arabidopsis by displacing substrates from the COP1 ubiquitin ligase

**DOI:** 10.1111/tpj.70071

**Published:** 2025-03-07

**Authors:** Laura Trimborn, Franziska Kuttig, Jathish Ponnu, Pengxin Yu, Kris R. Korsching, Patrick Lederer, Uriel Urquiza‐García, Matias D. Zurbriggen, Ute Hoecker

**Affiliations:** ^1^ Institute for Plant Sciences and Cluster of Excellence on Plant Sciences (CEPLAS), Biocenter University of Cologne Zülpicher Str. 47b 50674 Cologne Germany; ^2^ Institute of Synthetic Biology and CEPLAS University of Düsseldorf Universitätsstr. 1 40225 Düsseldorf Germany; ^3^ Present address: Department of Molecular Cell Biology Joseph Gottlieb Kölreuter Institute for Plant Sciences, Karlsruhe Institute of Technology Fritz‐Haber‐Weg 4 76131 Karlsruhe Germany

**Keywords:** COP1, cryptochromes, E3 ubiquitin ligase, light signaling, VP motif

## Abstract

In blue light, cryptochrome photoreceptors inhibit the key repressor of light signaling, the COP1/SPA ubiquitin ligase, to promote photomorphogenic responses. This inhibition relies on the direct interaction between COP1 and cryptochromes. Here, we analyzed the molecular mechanism of CRY1‐mediated inhibition of COP1. We show that the VP motif in the C‐terminal domain of CRY1 is essential for the COP1‐CRY1 interaction in Arabidopsis. Phenotypic analysis of transgenic Arabidopsis plants harboring a mutation in the VP motif reveals that the VP motif of CRY1 is required for blue light‐induced responses, such as seedling de‐etiolation and anthocyanin biosynthesis. Via its VP motif, CRY1 inhibits the interaction between COP1 and the COP1 substrate transcription factors PAP2 and HY5. Replacing the VP motif of CRY1 with that of the human COP1 interactor TRIB1 produces a functional photoreceptor in transgenic plants. Since HY5, PAP2 and CRY1 interact with COP1 through their respective VP motifs, our results demonstrate that CRY1 inhibits the activity of COP1 by competitively displacing substrates from COP1. Taken together with previous results showing VP‐dependent substrate displacement by photoactivated CRY2 and UVR8 photoreceptors, our results highlight the conservation of this mechanism across multiple photoreceptors.

## Significance Statement

Here, we demonstrate a critical molecular mechanism that leads to inactivation of the key repressor of light signaling in plants, the COP1 ubiquitin ligase, by the blue light photoreceptor cryptochrome 1 (CRY1). We reveal that CRY1 competitively displaces substrates from COP1, thereby promoting photomorphogenesis in blue light.

## INTRODUCTION

As sessile organisms, plants adapt their growth and development to changing light conditions to optimize their photosynthetic activity, survival and reproductive success. To perceive the intensity, wavelength, quality and direction of the incident light, plants have evolved a diverse set of photoreceptors: the red/far‐red responsive phytochromes, the blue‐light absorbing cryptochromes, phototropins and ZEITLUPE family and the UV‐B receptor UVR8 (Galvão & Fankhauser, 2015; Kami et al., 2010). After light perception, phytochromes, cryptochromes and UVR8 inactivate a key repressor of light signaling, the CONSTITUTIVELY PHOTOMORPHOGENIC1/SUPPRESSOR OF PHYTOCHROME A‐105 (COP1/SPA) complex (Ponnu & Hoecker, 2021). The COP1/SPA complex acts as a substrate receptor of a CULLIN4‐based E3 ubiquitin ligase (Cañibano et al., 2021; Chen et al., 2010). The function of COP1 depends on its interaction with SPA proteins (SPA1‐4) (Hoecker & Quail, 2001; Laubinger et al., 2004; Ordoñez‐Herrera et al., 2015; Saijo et al., 2003; Zhu et al., 2008). SPA proteins interact with COP1 to form a hetero‐tetrameric complex consisting of two COP1 and two SPA proteins (Zhu et al., 2008). Both COP1 and the SPA proteins contain a central coiled‐coil domain mediating homo‐ and heterodimerization and a C‐terminal WD40 repeat domain that interacts with substrates and photoreceptors. In their N‐termini, COP1 and SPA proteins differ; while COP1 has a RING‐finger domain, SPA proteins contain a kinase domain (Ponnu & Hoecker, 2021).

In darkness, the COP1/SPA complex mediates the polyubiquitination of positive regulators of light signaling, mainly transcription factors, leading to their degradation via the *26S* proteasome, thereby preventing photomorphogenesis (Ponnu & Hoecker, 2021). Examples include regulators of seedling de‐etiolation such as LONG HYPOCOTYL 5 (HY5) (Osterlund et al., 2000), flowering time regulators such as CONSTANS (CO) (Jang et al., 2008; Laubinger et al., 2006; Liu et al., 2008) or the anthocyanin biosynthesis activator PRODUCTION OF ANTHOCYANIN PIGMENT 1 (PAP1) and its homolog PAP2 (Maier et al., 2013). *cop1* and *spa* mutants, therefore, undergo constitutive photomorphogenesis in darkness and early flowering in short day (Laubinger et al., 2004; McNellis et al., 1994).

The COP1/SPA complex is inhibited by light through the actions of phytochromes, cryptochromes and UVR8, thereby allowing substrate transcription factors to accumulate and promote photomorphogenic responses. Multiple mechanisms are responsible for inhibiting the activity of the COP1/SPA complex in the light, including the nuclear exclusion of COP1 (Arnim & Deng, 1994; Balcerowicz et al., 2017; Osterlund & Deng, 1998; Pacín et al., 2014), the disruption of the COP1‐SPA1 interaction (Lian et al., 2011; Liu et al., 2011; Lu et al., 2015; Sheerin et al., 2015), the degradation of SPA1 and SPA2 proteins (Balcerowicz et al., 2011; Chen et al., 2015; Schenk et al., 2021) and the displacement of COP1 substrates from COP1 by UVR8 and crytochrome 2 (CRY2) (Lau et al., 2019; Ponnu et al., 2019).

The interaction between the cryptochrome photoreceptors CRY1 and CRY2 with the COP1/SPA complex is a key component of blue light‐mediated inhibition of COP1/SPA activity (Ponnu & Hoecker, 2022). *In planta* co‐immunoprecipitations have shown that both CRY1 and CRY2 interact with the COP1/SPA complex only in blue light and not in darkness (Holtkotte et al., 2017). Photoactivated CRY2 inactivates the COP1/SPA complex by competitively displacing substrates from the COP1‐WD repeat domain in blue light (Lau et al., 2019; Ponnu et al., 2019). This mechanism is based on the common COP1‐interacting motif, a valine‐proline (VP) motif, present in CRY2 and COP1 substrates. The VP motifs are characterized by the core sequence VPE/D‐Φ‐G (where Φ is designated for a hydrophobic residue), with an upstream stretch of four to five negatively charged amino acids (Holm et al., 2001; Uljon et al., 2016). Co‐crystallization of the human and Arabidopsis COP1‐WD with the mammalian COP1‐interactor Tribbles 1 (TRIB1) revealed a highly conserved VP‐binding pocket within the human and Arabidopsis COP1‐WD domains that directly binds the TRIB1 VP motif (Uljon et al., 2016). Recent studies further showed that the photoreceptor CRY2 also utilizes a VP motif to bind to COP1‐WD (Lau et al., 2019; Ponnu et al., 2019). In the light, photoactivated CRY2 competes with substrates, such as PAP2 and HY5, for binding to the VP binding pocket in the COP1‐WD repeat domain (Lau et al., 2019; Ponnu et al., 2019). By outcompeting these substrates, CRY2 displaces them from the COP1/SPA complex, thereby allowing their accumulation.

Like Arabidopsis COP1 (AtCOP1), human COP1 (hCOP1) functions as an E3 ubiquitin ligase. hCOP1 regulates various processes, such as cell proliferation, DNA repair and apoptosis, by promoting the degradation of key transcription factors (Song et al., 2020). Although CRYs also exist in humans they do not directly interact with hCOP1 and are regulators of circadian rhythms, DNA damage response and cancer progression (Michael et al., 2017). Thus, substrate recognition by COP1 is conserved between Arabidopsis and humans, while the regulation by CRY appears at least partially distinct.

Arabidopsis CRY1 and CRY2 are both blue light receptors but differ in their function, regulation and sequence. The CRY1 photoreceptor is mostly light‐stable, except for its destabilization in very high fluence rates of blue light, and plays a more prominent role in strong blue light (Lin et al., 1998; Miao et al., 2022). The CRY2 photoreceptor is light‐labile and undergoes rapid degradation in blue light, which contributes to its predominant function in low light conditions and its higher sensitivity towards blue light in comparison to CRY1 (Lin et al., 1998; Liu et al., 2020). CRY1 primarily functions in blue light‐induced seedling de‐etiolation, whereas CRY2 is mainly responsible for the induction of flowering under long‐day photoperiods, but also contributes to seedling de‐etiolation under low fluence rates of blue light. CRY1 and CRY2 also differ in their domains. While they are similar in the highly conserved N‐terminal Photolyase Homologous Region (PHR) which is responsible for light sensing, they differ in their carboxy terminus (CCT) which contributes to signal transduction by interacting with downstream proteins. The CCT domains of CRY1 (CCT1) and CRY2 (CCT2) are different in length and barely show any sequence similarity. Nevertheless, CRY1 – like CRY2 – utilizes its CCT domain to bind to the COP1‐WD repeat domain (Wang et al., 2001; Yang et al., 2001). Here, we show that the VP1 motif of CRY1 is essential for the CRY1‐COP1 interaction in Arabidopsis. Furthermore, we provide evidence for a VP‐mediated competition between CRY1 and the COP1 substrates HY5 and PAP2 as a mechanism to inhibit the COP1/SPA complex activity and thus to promote photomorphogenesis in Arabidopsis.

## RESULTS

### The VP1 motif of CRY1 is essential for the interaction between CRY1 and COP1 *in planta*

**Figure 1:**
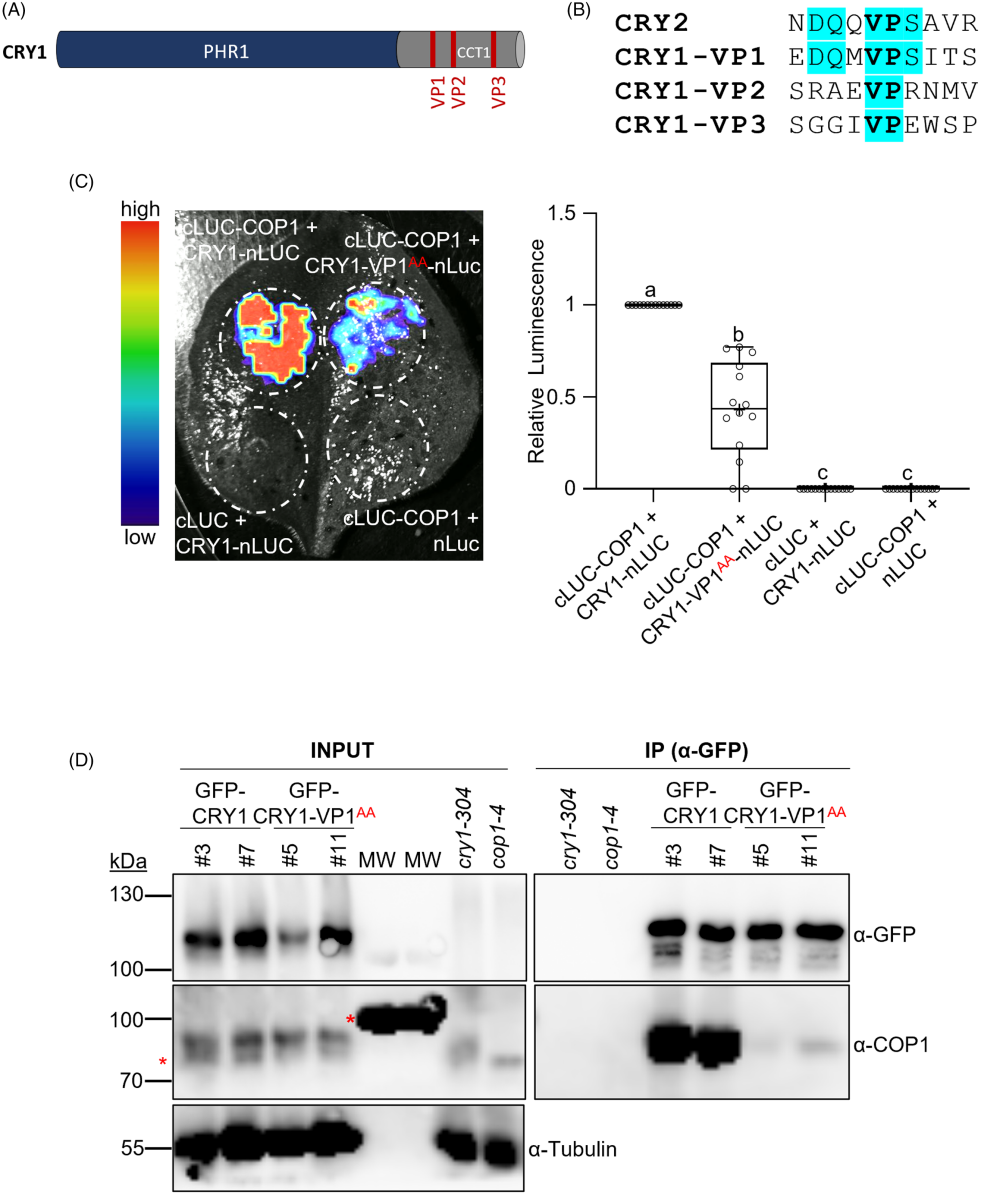
The first VP motif of CRY1 is essential for interaction with COP1 *in planta*. (A) Schematic representation of the CRY1 protein domains. N‐terminal photolyase homology region (PHR1) domain and CRY1 C‐terminal domain (CCT1), which harbors the three Valine Proline (VP) motifs. (B) Alignment of CRY2 and CRY1 VP motifs. Amino acids conserved in the alignment are highlighted in blue. (C) Luciferase (LUC) complementation imaging (LCI) assay showing that the interaction of COP1 and CRY1 depends on the VP1 motif of CRY1. COP1 was fused to the C‐terminal half of luciferase (cLUC) and CRY1 or CRY1 with VP1 mutated to Ala residues (CRY1‐VP1^AA^) were fused to the N‐terminal half of luciferase (nLUC). (Left) Image of luminescence detected in representative transfected *N. benthamiana* leaf. (Right) Relative luminescence signal in transfected leaves (*n* = 14), as measured using a CCD camera; upper and lower hinges of the boxplot correspond to the minimum and maximum value. Each individual replicate is plotted as a dot. The median is represented by a horizontal line within the box. The mean is represented by a ‘+’. Different letters indicate statistically significant differences (*P* ≤ 0.05) between two groups, estimated by one‐way ANOVA followed by Tukey's multiple comparisons. As control for transfection efficiency, GFP‐NLS‐GUS was co‐expressed in all transfections. GFP‐normalized luminescence (luminescence/GFP) is shown in Figure S2. The experiment was repeated twice with similar results. (D) Co‐immunoprecipitation of COP1 by GFP‐CRY1 or GFP‐CRY1‐VP1^AA^. Total proteins were extracted from transgenic *cry1‐304* mutant seedlings expressing GFP‐CRY1 or CRY1‐VP1^AA^. Seedlings were grown in darkness for 4 days and subsequently transferred to blue light (50 μmol m^−2^ s^−1^) for 1 h. Proteins were detected using α‐GFP, α‐COP1 and α‐Tubulin antibodies. Red asterisks mark nonspecific bands. The signal in the MW (molecular weight ladder) detected is due to a cross‐reaction with the COP1 antibody. The experiment was repeated twice with similar results.

The CRY1‐CCT domain contains three possible VP motifs which are conserved among CRY1 orthologs in angiosperms (Figure S1). The first one (VP1) has the highest sequence similarity with the single VP motif found in CRY2 (Figure 1A,B). We showed previously in yeast two‐hybrid (Y2H) studies that mutations in VP1 abolished the CRY1‐COP1 interaction, while mutations in VP2 or VP3 did not alter the CRY1‐COP1 interaction (Ponnu et al., 2019). The importance of VP1 for the CRY1‐COP1 interaction was further shown in co‐localization and FRET experiments (Ponnu et al., 2019) as well as in co‐crystallization and isothermal titration calorimetry (ITC) assays using recombinant COP1‐WD and a CRY1 VP1 peptide (Lau et al., 2019). To further verify the role of the CRY1 VP1 motif *in planta*, we mutated VP1 to alanine residues (CRY1‐VP1^AA^) by site‐directed mutagenesis and examined the interaction with COP1 using luciferase complementation imaging (LCI). For LCI, nLUC and cLUC fusion proteins were expressed in transfected *N. benthamiana* leaves and the obtained luminescence was imaged with a charge‐coupled device (CCD)‐based camera and quantified with Image J (Figure 1C). The CRY1‐VP1^AA^ mutation strongly reduced the interaction between COP1 and CRY1 (Figure 1C). To ensure that the observed differences in luminescence were not caused by differences in tobacco infiltration efficiency, GFP‐NLS‐GUS was co‐expressed as a transfection control and GFP fluorescence and luminescence signals of excised leaf discs were quantified using a plate reader (Figure S2). The similar GFP fluorescence in all samples indicates that the observed differences in luminescence were not caused by unequal transfection efficiency (Figure S2b,d). Consequently, also using this method of quantification, the results confirm that CRY1‐VP1^AA^ interacted much less strongly with COP1 than wild‐type CRY1 (Figure S2c,d).

Next, we tested whether YFP‐COP1 is able to recruit mCherry‐CCT1 into nuclear bodies (NBs), in the same way as it recruits mCherry‐CRY1 to NBs (Ponnu et al., 2019). Indeed, the mCherry‐CCT1 was recruited into NBs by YFP‐COP1, confirming previous results that CCT1 is sufficient for interaction with COP1 (Wang et al., 2001; Yang et al., 2001). The recruitment into NBs was dependent on the VP1 motif as mCherry‐CCT1‐VP1^AA^ did not form NBs in the presence of COP1 (Figure S3).

To analyze the relevance of VP1 for the CRY1‐COP1 interaction in Arabidopsis seedlings, we performed a co‐immunoprecipitation using transgenic lines that either overexpress GFP‐CRY1 (*35S:GFP‐CRY1*) or GFP‐CRY1‐VP1^AA^ (*35S:GFP‐CRY1‐VP1* ^*AA*^) in a *cry1‐304* mutant background. COP1 was effectively co‐immunoprecipitated by GFP‐CRY1 in blue light‐exposed seedlings, while GFP‐CRY1‐VP1^AA^ bound COP1 only very weakly (Figure 1D). Taken together, these results indicate that the VP1 motif of CRY1 is essential for the interaction between CRY1 and COP1 in Arabidopsis.

### The VP1 motif of CRY1 is essential for CRY1‐mediated seedling de‐etiolation and anthocyanin biosynthesis in blue light

**Figure 2:**
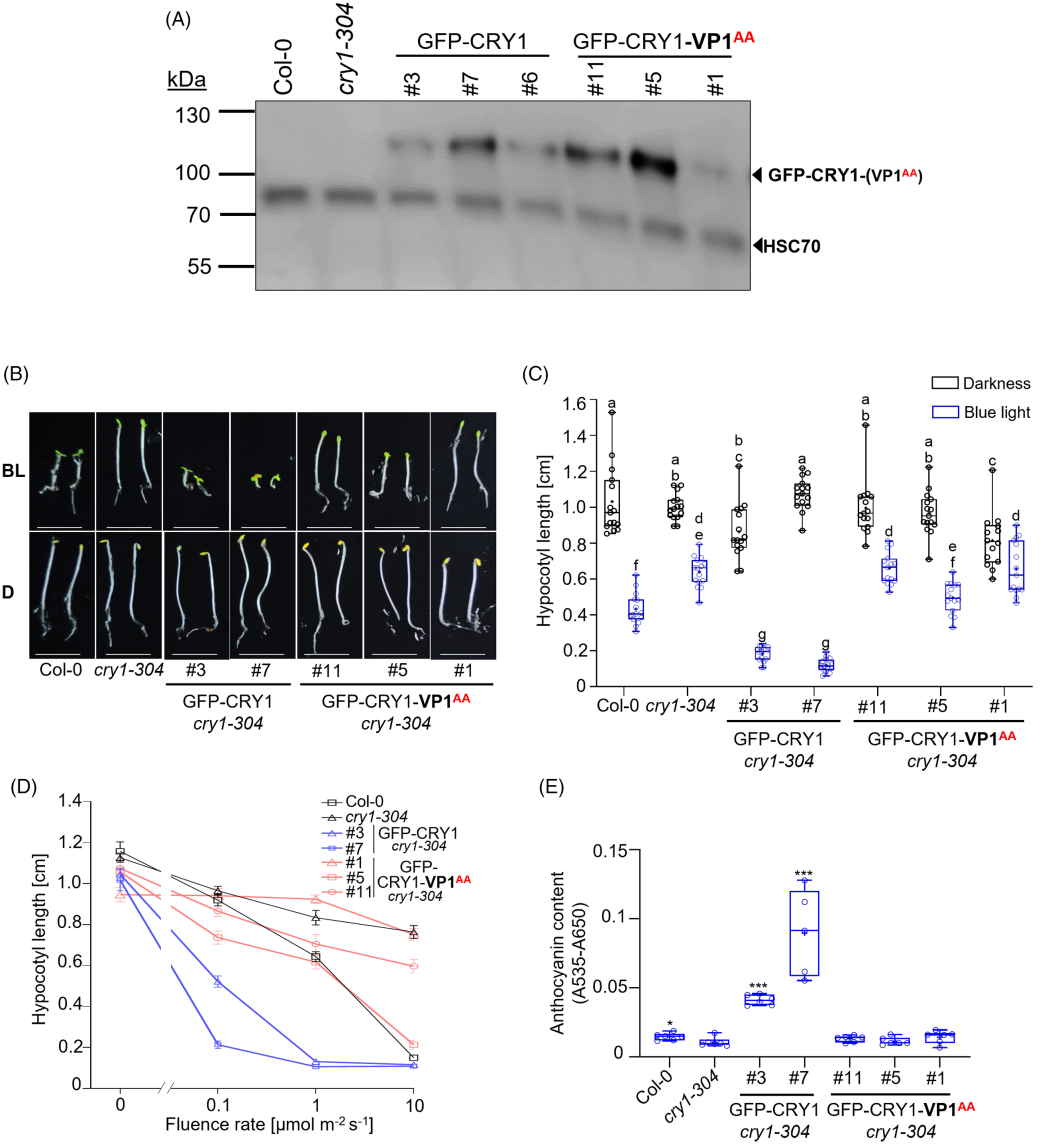
The first VP motif of CRY1 is essential for seedling de‐etiolation and anthocyanin biosynthesis in blue light. (A) Immunoblot analysis of transgenic *cry1‐304* seedlings expressing GFP‐CRY1 or GFP‐CRY1‐VP1^AA^ under the control of the *35S* promoter. Seedlings were grown in 2.5 μmol m^−2^ s^−1^ continuous blue light for 5 days. Line numbers (#) indicate independent transgenic lines. GFP‐tagged proteins were detected by an α‐GFP antibody. HSC70 levels detected by α‐HSC70 are shown as loading control. (B) Pictures of representative seedlings of 5‐day‐old *cry1‐304* mutants expressing GFP‐CRY1 or CRY1‐VP1^AA^ when grown in darkness (D) or 2.5 μmol m^−2^ s^−1^ continuous blue light (BL) compared with WT (Col‐0) and *cry1‐304*. Scale bar = 0.5 cm. (C) Hypocotyl length of 5‐day‐old seedlings of the indicated genotypes grown as in (B). Box plots are shown for n > 20 seedlings. Differences between genotypes were estimated using two‐way ANOVA followed by Tukey's multiple comparison. Different letters indicate statistically significant differences (*P* ≤ 0.05). (D) Fluence‐dose response curves of the same genotypes as in (A) when grown for 4 days in darkness or at the indicated fluence rates of continuous blue light. Data show the mean of at least 15 seedlings ± SEM. (E) Anthocyanin content of 7‐day‐old seedlings of the same genotypes as in (A + B) when grown in 10 μmol m^−2^ s^−1^ continuous blue light. Asterisks indicate a significant difference in anthocyanin levels between the respective genotype and *cry1‐304*, analyzed using Student's *t*‐test with *P = 0.01–0.05 and ***P < 0.001. (A–E) All experiments were performed at least three times. Representative results are shown here.

To investigate whether the VP1 motif of CRY1 is important for CRY1 biological function in Arabidopsis, we performed a phenotypic analysis of the GFP‐CRY1 and GFP‐CRY1‐VP1^AA^ overexpression lines. Among these lines, GFP‐CRY1 and GFP‐CRY1‐VP1^AA^ protein levels varied, but we selected several transgenic lines for each transgene that showed comparable protein levels (Figure 2A). Expression of GFP‐CRY1 in the *cry1‐304* mutant complemented the elongated‐hypocotyl phenotype of *cry1‐304* in blue light (Figure 2B,C). These lines showed an even shorter hypocotyl in blue light than the wild type, which is indicative of the overexpression of CRY1. In contrast, transgenic *cry1‐304* lines expressing GFP‐CRY1‐VP1^AA^ did not significantly differ from the *cry1‐304* mutant (Figure 2B,C). These results indicate that the VP1^AA^ mutation strongly compromised CRY1 activity. Fluence dose response curves confirmed this conclusion: CRY1‐VP1^AA^‐expressing lines were insensitive or hyposensitive to blue light (Figure 2D). Only line #5 which accumulated very high GFP‐CRY1‐VP1^AA^ protein levels (Figure 2A) showed some reduction in hypocotyl elongation when compared to the *cry1‐304* progenitor (Figure 2D). This response was, however, still dramatically lower than that of wild‐type GFP‐CRY1‐expressing lines. We further examined the anthocyanin level in these transgenic lines. The expression of GFP‐CRY1, but not of GFP‐CRY1‐VP1^AA^, complemented the *cry1‐304* mutant phenotype (Figure 2E). Taken together, these results demonstrate that the VP1 motif in CRY1 is essential for CRY1‐mediated de‐etiolation and anthocyanin biosynthesis in response to blue light.

### CRY1 inhibits the interaction of COP1 with its substrate PAP2

As the VP1 motif of CRY1 is essential for the interaction between CRY1 and COP1 and functions in blue light signaling, we hypothesized that CRY1 is able to displace transcription factors from COP1 in a similar manner as CRY2 and UVR8 do (Lau et al., 2019; Ponnu et al., 2019). We tested this hypothesis using the COP1 substrates PAP2 and HY5 because it was shown earlier that both use VP motifs to bind to COP1 (Holm et al., 2001; Lau et al., 2019; Maier et al., 2013; Ponnu et al., 2019).

**Figure 3:**
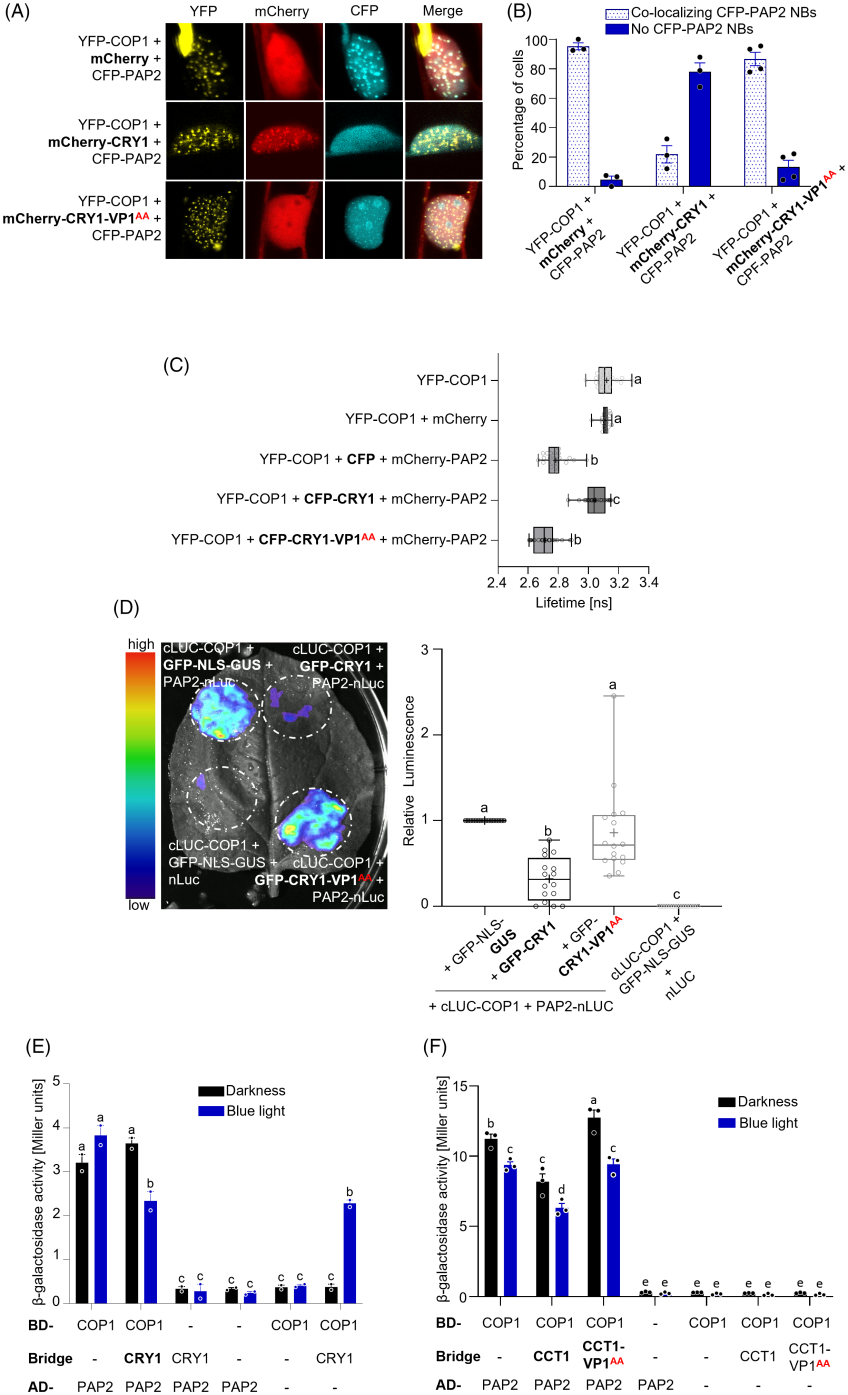
CRY1 displaces PAP2 from COP1. (A) Representative confocal images of leek epidermal cells co‐expressing the indicated fusion proteins after particle bombardment. (B) Percentage of cells that formed mCherry‐PAP2 nuclear bodies (NBs) co‐localizing with YFP‐COP1 when indicated fusion proteins were co‐expressed in leek epidermal cells. The experiment was repeated at least three times, with 25 cells being evaluated for each combination in each experiment. Bars show the mean ± SEM. Dots represent the mean value of the independent experiments. (C) FRET‐FLIM assays showing the lifetime of the donor fluorophore (YFP‐COP1) measured inside the whole nucleus of leek cells after particle bombardment. Box plots are shown for *n* > 20 cells measured in three independent bombardments. Left and right hinges of the boxplot correspond to the minimum and up to the maximum value. Each individual replicate is plotted as a dot. The median is represented by a horizontal line within the box. The mean is represented by a ‘+’. (D) LCI assay showing the interaction of COP1 and PAP2 in presence of GFP‐CRY1, GFP‐CRY1‐VP1^AA^ or GFP‐NLS‐GUS (neg. control). COP1 was fused to cLUC and PAP2 to nLUC. Left panel: Image of luminescence detected in representative transfected *N. benthamiana* leaf. Right panel: Relative luminescence signal in transfected leaves (*n* = 16), as measured using a CCD camera. As transfection control, TagRFP was co‐expressed. Tag‐RFP‐normalized luminescence (luminescence/TagRFP) is shown in Figure S5. The experiment was repeated twice with similar results. (E, F) Yeast three‐hybrid (Y3H) assays with COP1 as bait and PAP2 as prey in presence or absence of (E) CRY1 or (F) CCT1 or CCT1‐VP1^AA^. Co‐transformed yeast cells grew in darkness for 2 days (darkness) or 1 day in darkness, followed by exposure to 50 μmol m^−2^ s^−1^ blue light for 1 day (blue light). BD, DNA binding domain; AD, activation domain. The experiments were repeated at least twice with similar results. (C–E) Statistical differences were estimated using (C–D) one‐way or (E) two‐way ANOVA followed by Tukey's multiple comparison. Different letters indicate statistically significant differences (*P* ≤ 0.05).

To investigate whether CRY1 competes with PAP2 for binding to COP1 *in planta* we performed a co‐localization competition assay by expressing YFP‐COP1 and CFP‐PAP2 in the presence of co‐expressed mCherry‐CRY1 or mCherry (as negative control) in bombarded leek epidermal cells. In the presence of mCherry, YFP‐COP1 and CFP‐PAP2 co‐localized in nuclear bodies (NBs) in more than 90% of the transfected cells (Figure 3A,B). Here, YFP‐COP1 recruited CFP‐PAP2 into NBs because CFP‐PAP2 only localized to NBs when YFP‐COP1 was co‐expressed, which is in line with previous co‐localization experiments (Figure S4) (Maier et al., 2013). This is a strong indication for an interaction between COP1 and PAP2. When mCherry‐CRY1 was co‐expressed with YFP‐COP1 and CFP‐PAP2, mCherry‐CRY1 disrupted the NB formation of CFP‐PAP2 in the large majority of tested cells (Figure 3A,B). Instead, YFP‐COP1 co‐localized with mCherry‐CRY1 in NBs. When mCherry‐CRY1‐VP1^AA^ was co‐expressed with YFP‐COP1 and CFP‐PAP2, a disruption of COP1‐PAP2 co‐localization was not observed. These results indicate that CRY1 disrupts the COP1‐PAP2 co‐localization in a VP1‐dependent fashion.

To corroborate these results, we performed a FRET‐FLIM analysis on co‐bombarded leek cells that expressed YFP‐COP1, mCherry‐PAP2 and CFP‐CRY1 (Figure 3C). YFP in YFP‐COP1 exhibited a lifetime of approx. 3.1 ns, which is in agreement with previously published values for YFP (Borst et al., 2010; Kwaaitaal et al., 2010; Ponnu et al., 2019). Co‐expression of mCherry did not change the YFP‐COP1 lifetime. The lifetime of YFP‐COP1 was significantly reduced in the presence of co‐expressed mCherry‐PAP2, confirming an interaction between COP1 and PAP2 *in planta*. When CFP‐CRY1 was co‐expressed with YFP‐COP1 and mCherry‐PAP2, the YFP‐COP1 lifetime was significantly higher when compared to co‐expression of CFP. It was almost as high as in those cells expressing YFP‐COP1 alone. This indicates that CFP‐CRY1 disrupts the interaction between YFP‐COP1 and mCherry‐PAP2 (Figure 3C). In contrast, when the mutant version CFP‐CRY1‐VP1^AA^ was co‐expressed with YFP‐COP1 and mCherry‐PAP2, the lifetime of YFP‐COP1 did not change when compared to co‐expression of YFP‐COP1, mCherry‐PAP2 and CFP. This demonstrates that CFP‐CRY1‐VP1^AA^ was not able to disrupt the COP1‐PAP2 interaction (Figure 3C). This finding confirms that the effect of CRY1 on the COP1‐PAP2 interaction depends on the VP1 motif of CRY1.

To further test our hypothesis, we performed an LCI assay (Figure 3D). cLUC‐COP1 and PAP2‐nLUC were co‐expressed with GFP‐CRY1, GFP‐CRY1‐VP1^AA^ or GFP‐NLS‐GUS (as negative control). A strong luminescence signal was observed when GFP‐NLS‐GUS was co‐expressed, indicating an interaction between COP1 and PAP2. The co‐expression of GFP‐CRY1 reduced the luminescence signal by about 50%, indicating that CRY1 reduces the COP1‐PAP2 interaction (Figure 3D). In contrast, the co‐expression of GFP‐CRY1‐VP1^AA^ did not significantly reduce the COP1‐PAP2 interaction, confirming that the effect of CRY1 on the COP1‐PAP2 interaction depends on the VP1 motif (Figure 3D). To again ensure that the observed differences in the luminescence signal were not influenced by discrepancies in tobacco infiltration efficiencies, Tag‐RFP was co‐expressed as transfection control and RFP fluorescence, GFP fluorescence and luminescence signals of excised leaf discs were quantified using a plate reader (Figure S5). Similar RFP levels in all samples indicate that the observed differences in luminescence were not the result of variations in transfection efficiencies (Figure S5a). Moreover, both GFP‐CRY1 and GFP‐CRY1‐VP1^AA^ showed similar GFP fluorescence; thus, the VP1^AA^ mutation did not affect CRY1 levels (Figure S5b). RFP‐normalized luminescence signals confirm that CRY1, but not CRY1‐VP1^AA^, reduced the COP1‐PAP2 interaction (Figure S5c,d).

Since COP1 interacts with the CCT domain of CRY1 (Wang et al., 2001; Yang et al., 2001) (Figure S3) we furthermore tested in a co‐localization competition assay whether the CCT1 domain is sufficient to inhibit the COP1‐PAP2 co‐localization (Figure S6A,B). CFP‐CCT1 disrupted the NB formation of mCherry‐PAP2 in the majority of the tested cells, but not as efficiently as CFP‐CRY1 (Figure S6A,B).

Additionally, we performed an LCI assay. The co‐expression of GFP‐NLS‐CCT1 did not reduce the COP1‐PAP2 interaction (Figure S6C). Previous studies showed that CRY1 needs to oligomerize via the PHR domain to be functional (Liu et al., 2020; Sang et al., 2005). We therefore hypothesized that the GFP‐NLS‐CCT1 protein was not able to displace PAP2 from COP1 because it lacks a dimerization domain. We thus repeated the experiment and fused CCT1 to GUS as artificial dimerization domain, a fusion protein that was previously shown to be functional (Yang et al., 2000). Co‐expression of GFP‐NLS‐GUS‐CCT1 indeed reduced the COP1‐PAP2 interaction (Figure S6D). This reduction was not observed when GFP‐NLS‐GUS‐CCT1‐VP1^AA^ was co‐expressed, confirming that the displacement of PAP2 from COP1 relies on the VP1 motif of CCT1.

Additionally, we performed yeast three‐hybrid assays comparing the interaction strength of COP1 and PAP2 in the presence and absence of CRY1 (Figure 3E). When CRY1 was co‐expressed (as ‘bridge protein’), the COP1‐PAP2 interaction was strongly reduced in blue light but not in darkness, suggesting that the inhibition of the COP1‐PAP2 interaction by CRY1 in yeast is blue light‐dependent. Notably, BD‐COP1/CRY1/AD‐PAP2 co‐expression resulted in a similar β‐galactosidase activity as the expression of the BD‐COP1/CRY1 negative control, indicating a high autoactivation of the reporter gene by CRY1 bound to BD‐COP1. This suggests that CRY1 fully disrupts the COP1‐PAP2 interaction in blue light. To investigate the role of the VP1 motif for the CRY1‐mediated inhibition of the COP1‐PAP2 interaction, we performed an additional Y3H assay co‐expressing CCT1 or CCT1‐VP1^AA^ as bridge proteins, which do not autoactivate the reporter gene (Figure 3F). Expression of the CCT1 domain reduced the COP1‐PAP2 interaction in blue light and darkness (Figure 3F). Here, a blue light‐dependency is not expected because CCT1 lacks the chromophore‐binding PHR domain of CRY1. In contrast, when CCT1‐VP1^AA^ was co‐expressed as bridge protein, the interaction between COP1‐PAP2 was unaffected (Figure 3F).

Taken together, these results show that CRY1 uses its VP1 motif to interact with COP1, thereby displacing PAP2 from COP1. In Y3H and co‐localization studies, CCT1 was able to displace PAP2 from COP1, while LCI assays required a fusion of GUS to CCT1 for CCT1 to inhibit the PAP2‐COP1 interaction. Also in co‐localization assays, full‐length CRY1 was more effective than CCT1. This implies that oligomerization of CRY1 may promote displacement of PAP2 from COP1. Moreover, full‐length CRY1 may provide cooperative binding between CRY1‐CCT1 and COP1, as it was shown for CRY2 (Lau et al., 2019).

### CRY1 inhibits the interaction of COP1 with its substrate HY5

**Figure 4:**
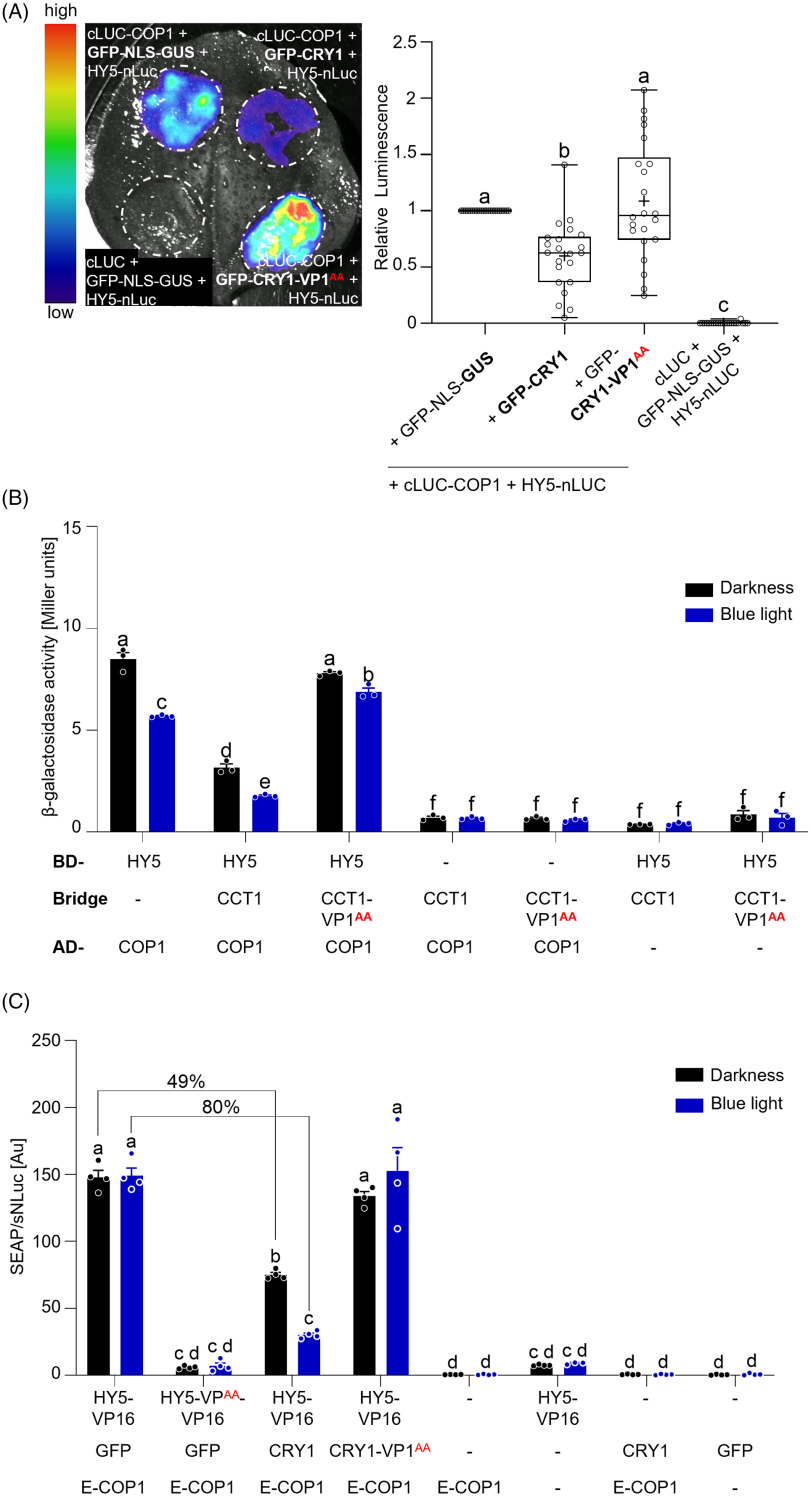
CRY1 displaces HY5 from COP1. (A) LCI assay showing the COP1‐HY5 interaction in presence of GFP‐CRY1, GFP‐CRY1‐VP1^AA^ or GFP‐NLS‐GUS (neg. control). COP1 was fused to cLUC and HY5 to nLUC. Left panel: Image of luminescence detected in representative transfected *N. benthamiana* leaf. Right panel: Relative luminescence signal in transfected leaves (*n* = 22), as measured using a CCD camera. As transfection control, TagRFP was co‐expressed. Tag‐RFP‐normalized luminescence (luminescence/TagRFP) is shown in Figure S7. (B) Y3H assays with COP1 as prey and HY5 as bait in presence or absence of CCT1 or CCT1‐VP1^AA^. Co‐transformed yeast cells grew in darkness for 2 days (darkness) or for 1 day in darkness, followed by exposure to 50 μmol m^−2^ s^−1^ blue light for 1 day (blue light). Dots represent the values of biological replicates. BD, DNA binding domain; AD, activation domain. (C) Mammalian three‐hybrid (M3H) assay showing the COP1‐HY5 interaction in presence of GFP (control), CRY1 or CRY1‐VP1^AA^. CHO‐K1 cells were transiently co‐transfected with the human placental secreted alkaline phosphatase (SEAP) reporter plasmid and the normalization plasmid constitutively expressing secreted NanoLuciferase (sNLuc). In addition, HY5 fused to the herpes simplex virus‐derived transactivation domain (VP16), COP1 fused to the DNA‐binding domain Erythromycin (E) and GFP, CRY1 or CRY1‐VP1^AA^ were co‐transfected. Cells were kept in the darkness or illuminated with blue light (10 μmol m^−2^ s^−1^) for 24 h. SEAP and sNLuc values were determined in the cell culture medium. Dots represent the values of biological replicates. The reduction percentage is shown above the bars. (A–C) Statistical differences were estimated using one‐ (A) or two‐way (B‐C) ANOVA followed by Tukey's multiple comparison. Different letters indicate statistically significant differences (*P* ≤ 0.05). (B, C) Error bars indicate SEM. All experiments were repeated twice with similar results.

To investigate whether CRY1 competes with other COP1 substrates for binding to COP1, we analyzed how CRY1 affects the COP1‐HY5 interaction in LCI and Y3H assays (Figure 4). Co‐expression of GFP‐CRY1, but not of GFP‐CRY1‐VP1^AA^, with cLUC‐COP1 and HY5‐nLUC caused a reduction of the luminescence signal compared to co‐expression of the GFP‐NLS‐GUS negative control (Figure 4A). Normalization of the luminescence signal to the Tag‐RFP infiltration control confirmed this conclusion (Figure S7). This indicates that CRY1 also reduces the COP1‐HY5 interaction in a VP1‐dependent fashion. Consistent with this, CCT1 abolished the COP1‐HY5 interaction in the Y3H assay in a VP1‐dependent fashion (Figure 4B).

We subsequently employed a three‐hybrid system in mammalian cell cultures (M3H) to investigate whether CRY1 can displace HY5 from COP1 in the absence of other plant proteins (Figure 4C) (Jang et al., 2023; Müller et al., 2013). In M3H, the bait protein is fused to the DNA‐binding erythromycin repressor protein (abbreviated ‘E’), while the prey protein is fused to the transcription activation domain VP16. If bait and prey interact, then the reporter gene *SEAP* (human placental secreted alkaline phosphatase) is expressed (Figure S8A). A third ‘bridge’ protein can be co‐expressed. As transfection control, the constitutively expressed *secNLUC* gene is co‐expressed and used for normalization. First, we established the interaction between COP1 and CRY1 in the mammalian two‐hybrid (M2H) system (Figure S8A). Co‐expression of E‐COP1 and CRY1‐VP16 led to high SEAP activity in mammalian cells illuminated with blue light, indicating an interaction of COP1 and CRY1 in blue light (Figure S8B). In contrast, co‐expression of E‐COP1 and CRY1‐VP1^AA^‐VP16 resulted in strongly reduced SEAP levels, confirming the importance of the VP1 motif of CRY1 for the interaction with COP1 also in this system (Figure S8B). Next, we established the COP1‐HY5 interaction (Figure 4C). In line with previous publications (Holm et al., 2001; Lau et al., 2019), the COP1‐HY5 interaction depended on the VP motif in HY5 (Figure 4C) (Holm et al., 2001; Lau et al., 2019). In blue light, the co‐expression of CRY1 reduced the COP1‐HY5 interaction by about 80% compared to the co‐expression of GFP (as negative control). Although CRY1 also reduced the COP1‐HY5 interaction in darkness, the inhibitory effect of CRY1 was much stronger in blue light than in darkness. This is consistent with the strong interaction between CRY1 and COP1 in blue light (Figure S8). How CRY1 inhibits the COP1‐HY5 interaction in dark‐exposed mammalian cells is not quite understood. The mutant version CRY1‐VP1^AA^ neither reduced the COP1‐HY5 interaction in blue light nor in darkness, confirming the VP1‐dependency of the observed CRY1‐mediated displacement of HY5 from COP1.

In summary, these findings demonstrate that CRY1 utilizes its VP1 motif to compete with PAP2 and HY5 for binding to COP1, thereby disrupting the interaction between COP1 and these transcription factors.

### Chimeric proteins of Arabidopsis CRY1 and human TRIB1 VP motif are functional in blue light signaling

**Figure 5:**
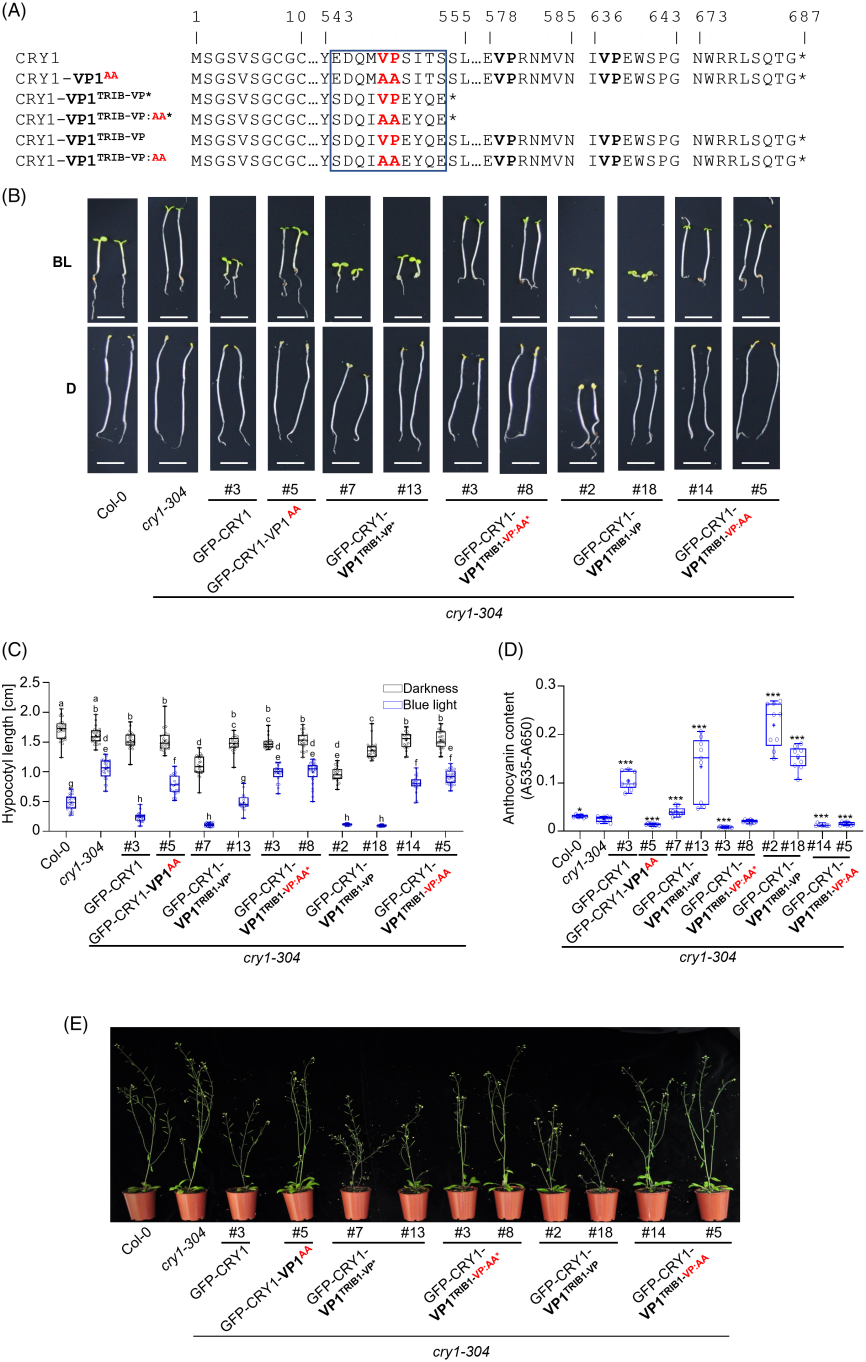
Chimeric proteins of Arabidopsis CRY1 and human TRIB1 VP motif are functional in blue light signaling. (A) Amino acid sequence alignment of CRY1, CRY1‐VP1^AA^ and CRY1‐TRIB1 chimeric proteins. Asterisks indicate a stop codon. The framed amino acids are the core VP motif. Numbers above the amino acid sequence refer to the position of the amino acid within the respective protein sequence. (B–D) Phenotypic analysis of transgenic *cry1‐304* mutants expressing the indicated CRY1 mutant variants fused to GFP. Box plots are shown for *n* > 20 seedlings. The phenotypic analyses were repeated twice with similar results. (B, C) Representative pictures (B) and hypocotyl length (C) of 7‐day‐old seedlings of the indicated genotypes grown in darkness (D) or 2.5 μmol m^−2^ s^−1^ continuous blue light (BL). Differences between genotypes were estimated using two‐way ANOVA followed by Tukey's multiple comparison. Different letters indicate statistically significant differences (*P* ≤ 0.05) (Scale bar = 0.5 cm). (D) Anthocyanin content of 7‐day‐old transgenic seedlings of the indicated genotypes grown in 10 μmol m^−2^ s^−1^ continuous blue light. Asterisks indicate a significant difference in anthocyanin levels between the respective genotype and *cry1‐304*, analyzed using Student's *t*‐test with *P = 0.01–0.05 and ***P < 0.001. (E) Phenotype of 5‐week‐old adult plants grown under long‐day conditions. All transgenes were expressed under the control of the 35S promoter.

If this displacement mechanism is indeed important for *in vivo* photomorphogenesis, then replacing the VP1 core sequence in CCT1 with the VP core sequence of another COP1‐interacting protein should maintain CRY1 function in photomorphogenesis. To test this hypothesis, we generated constructs to express chimeric proteins in which the VP1 core motif of CRY1 was replaced by the VP core motif of human TRIB1. Crystallography studies have shown that the VP motif of the human TRIB1 protein directly interacts with the Arabidopsis COP1‐WD40 domain (Uljon et al., 2016). Since it is a human protein, it is sufficiently orthogonal in the plant background to not have any other known functions in plants. Moreover, the VP motif of TRIB1 was previously shown to be active in a UVR8 context (Lau et al., 2019). We generated two types of CRY1‐TRIB1 fusion constructs: in CRY1‐VP1^TRIB1*^, the replacement of the VP1 core was followed by a stop codon (*), thus truncating CCT1. In CRY1‐VP1^TRIB1^, the VP1 core of CRY1 was replaced by the VP core of TRIB1, thus maintaining the CCT1 sequence C‐terminal to the VP1 site (Figure 5A). Both fusion proteins interacted with COP1 in Y2H assays with a similar apparent strength as CRY1 (Figure S9). We expressed these chimeras in the *cry1‐304* mutant background and analyzed the phenotypes of selected transgenic lines that express the chimeric proteins at comparable levels (Figure 5B–E; Figure S10). Both *GFP‐CRY1‐VP1* ^TRIB1*^ and *GFP‐CRY1‐VP1* ^*TRIB1*^ transgenes complemented the hypocotyl and anthocyanin phenotypes of the *cry1‐304* mutant under blue light, indicating that the chimeric proteins are functional in Arabidopsis (Figure 5B–E). Interestingly, line #7 of GFP‐CRY1‐VP1^TRIB1^* and both lines (#2 and #18) of GFP‐CRY1‐VP1^TRIB1^ had a significantly shorter hypocotyl and partially open cotyledons when grown in darkness, suggesting a weak light‐independent activity of the chimeric proteins. This suggests that the proposed ‘closed conformation’ of CRY1 in darkness (Yu et al., 2007) may not be fully maintained in the chimeric photoreceptors. When analyzing the phenotype of adult plants, overexpression of CRY1 led to a dwarfed stature of the inflorescence (Figure 5E). A similar dwarfism was observed in plants overexpressing the CRY1‐TRIB1 fusions.

To confirm that the observed complementation of the *cry1‐304* mutant phenotype was indeed due to the introduction of the TRIB1‐VP motif, we mutated the VP of the VP core motif of TRIB1 to alanine residues (CRY1‐VP1^TRIB1‐VP:AA*^: mutated TRIB1 VP motif followed by a stop codon and CRY1‐VP1^TRIB1‐VP:AA^: mutated TRIB1 VP motif, full‐length CCT1) (Figure 5A). We overexpressed these fusion proteins in the *cry1‐304* mutant background and included them in the phenotypic analysis (Figure 5B–E; Figure S11). Lines expressing GFP‐CRY1‐VP1^TRIB1‐VP:AA*^ or GFP‐CRY1‐VP1^TRIB1‐VP:AA^ showed no complementation of the *cry1‐304* mutant hypocotyl phenotype under blue light. Thus, CRY1‐VP1^TRIB1^ activity was strongly dependent on the VP motif of TRIB1. Similarly, these lines did not show a restoration of anthocyanin accumulation in the *cry1‐304* mutant in blue light (Figure 5D). In addition, the adult plants expressing GFP‐CRY1‐VP1^TRIB1‐VP:AA*^ or GFP‐CRY1‐VP1^TRIB1‐VP:AA^ did not show the dwarfism that was observed in plants overexpressing the CRY1‐TRIB1 fusions without a mutation in the VP (Figure 5E; Figure S11D). Taken together, these analyses reveal that the complementation observed in both CRY1‐VP1^TRIB1^ lines depends on the presence of the VP motif of TRIB1. Thus, these results show that the TRIB1 VP core motif can functionally replace the CRY1 VP1 core motif.

## DISCUSSION

**Figure 6:**
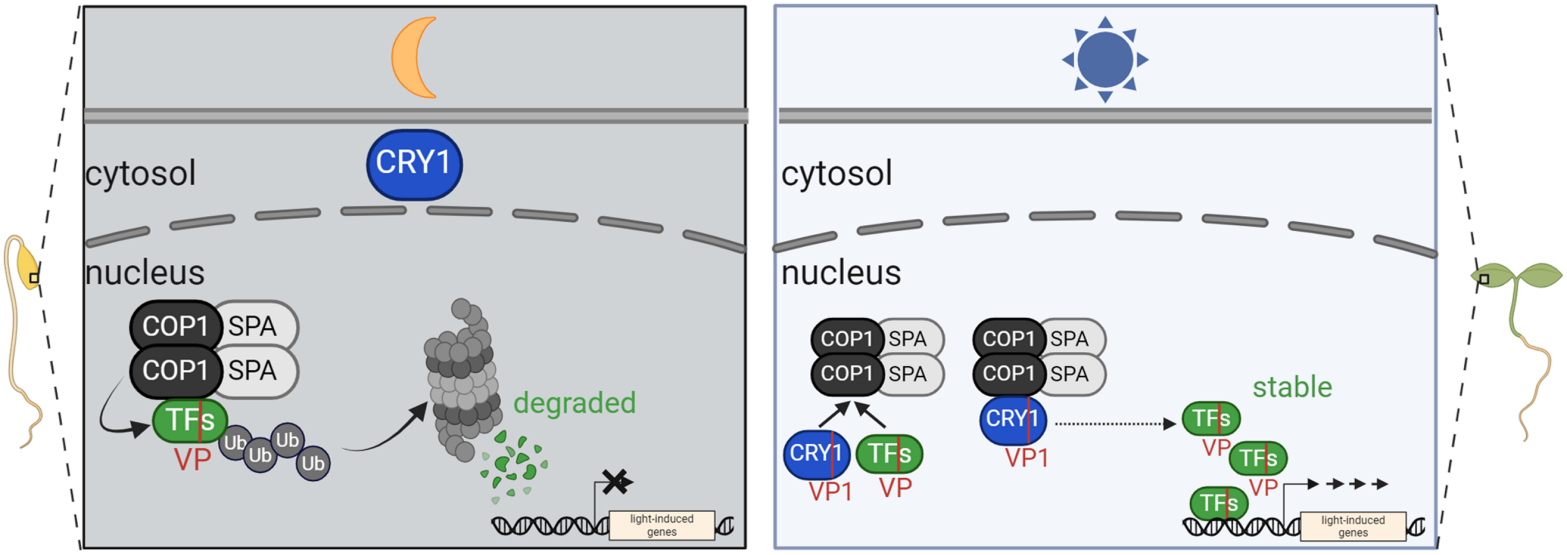
VP‐mediated inhibition of the COP1/SPA complex by CRY1. (Left) In darkness, the transcription factors (TFs), such as HY5 and PAP2, bind the COP1‐WD repeat domain via their VP motif. This leads to their degradation via the *26S* proteasome. (Right) Upon blue light exposure, CRY1 is activated, moves to the nucleus and competes with the TFs for binding to COP1. By binding to COP1 via its VP1 motif, CRY1 outcompetes the TFs, which leads to their stabilization and the induction of light‐responsive genes which then promote light responses such as seedling de‐etiolation and anthocyanin biosynthesis. Created in BioRender. Hoecker, U. (2023) BioRender.com/o73l502.

The COP1/SPA complex functions as a central repressor of light signaling in darkness by targeting and polyubiquitinating transcription factors that promote photomorphogenesis (Ponnu & Hoecker, 2021). These transcription factors, such as HY5 and PAP2, share a VP motif that is bound by COP1 (Holm et al., 2001; Lau et al., 2019; Maier et al., 2013; Ponnu et al., 2019). The interaction between the photoreceptors CRY1 and CRY2 and the COP1/SPA complex in blue light (Holtkotte et al., 2017) plays a crucial role in the blue light‐mediated inactivation of the COP/SPA complex. This inhibition allows for a broad range of photomorphogenic responses, such as seedling de‐etiolation and anthocyanin accumulation (Ponnu, 2020). Here, we revealed a critical molecular mechanism of CRY1‐mediated inhibition of the COP1/SPA complex (Figure 6). We demonstrated that CRY1 uses its VP1 motif to competitively displace VP‐containing COP1‐substrates from binding to COP1. It was shown previously that CRY2 and the UV‐B photoreceptor UVR8 also utilize the VP‐mediated competition mechanism to displace transcription factors from the COP1‐WD domain in blue light or UV‐B light, respectively (Lau et al., 2019; Ponnu et al., 2019). Taken together, these findings show that multiple photoreceptors use their VP motifs to inhibit COP1 by displacing substrates from binding to COP1‐WD.

We have shown by LCI assays in tobacco and co‐immunoprecipitation in Arabidopsis that the CRY1‐COP1 interaction depends on the VP1 motif in the CCT domain of CRY1 *in planta*. These findings are consistent with previous studies which utilized co‐crystallization and ITC analyses of recombinant COP1‐WD and a CRY1 peptide, Y2H and co‐localization experiments to show that CRY1 binds COP1 via its VP1 motif (Lau et al., 2019; Ponnu et al., 2019). The biological relevance of the VP1 motif for CRY1 activity is supported by the phenotype of our transgenic lines that express CRY1‐VP1^AA^ as these lines failed to complement the *cry1‐304* mutant phenotype in blue light. Consistent with this, a previous study found that the *hy4‐9* mutant, which contains a proline‐to‐leucine mutation in the VP1 motif of CRY1, showed no de‐etiolation and no anthocyanin accumulation in blue light (Ahmad et al., 1995).

The CRY1 VP1‐dependent interaction with COP1 suggests that CRY1 utilizes the VP‐mediated displacement mechanism to outcompete COP1/SPA substrates from the WD repeat domain of COP1, as previously hypothesized (Müller & Bouly, 2015; Lau et al., 2019; Ponnu et al., 2019). Indeed, we showed via various *in planta* assays, Y3H assays and M3H assays that CRY1 displaces the substrates PAP2 and HY5 from COP1. Moreover, we showed that the ability of CRY1 to displace PAP2 or HY5 from COP1 relies on the presence of the VP1 motif in CRY1. In a previous study, it was shown via ITC assays that the binding affinity of the CRY1 VP peptide for recombinant COP1‐WD is higher compared to a HY5 VP peptide (Lau et al., 2019), supporting our finding that CRY1 outcompetes substrates from the COP1‐WD domain. Protein–protein interaction studies showed that full‐length UVR8 and CRY2 showed higher binding affinity for COP1 than the respective VP‐containing peptides, suggesting a light‐induced cooperative binding mechanism involving the VP motif and additional interaction surfaces (Lau et al., 2019). A similar cooperative binding may exist for CRY1. Indeed, we found at least in co‐localization studies that full‐length CRY1 was more effective in inhibiting the COP1‐PAP2 interaction than CCT1. Cooperative binding may also involve SPA proteins in the COP1/SPA complex. In particular, we found previously, that the interaction between SPA1 and CRY1 was not fully dependent on VP1 and thus involves additional domains in SPA1 (Ponnu et al., 2019).

We further investigated the molecular mechanism of CRY1 action by replacing the VP1 motif of CRY1 with a foreign COP1‐binding motif, that is, from the human TRIB1 protein which was previously shown to bind the Arabidopsis COP1‐WD domain through its VP core sequence (Uljon et al., 2016). Indeed, the chimeric CRY1‐TRIB1 proteins interacted with COP1 and complemented the *cry1‐304* mutant phenotype in a VP‐dependent fashion. This gain‐of‐function approach demonstrates that an alternative, effective VP motif in CRY1‐CCT1 is able to inhibit COP1 activity. Similarly, *uvr8* null mutants that expressed chimeric proteins in which the UVR8 core domain was fused to the VP‐motif of TRIB1 were functional under UV‐B light (Lau et al., 2019). Since the CRY1‐VP1^TRIB1^* protein was fully functional in blue light, the results suggest that amino acids C‐terminal to the VP motif are not necessary for CRY1 activity. Similarly, the homologous NC80 motif in CRY2 was sufficient for CRY2 activity (Yu et al., 2007).

While the VP1 motif in CRY1 is clearly involved in substrate displacement from COP1, the putative VP2 and in particular VP3 are also well‐conserved among angiosperm CRY1 sequences. Mutations in VP2 and VP3 did not impact the CRY1‐COP1 interaction (Ponnu et al., 2019). Thus, VP2 and VP3 might be important for CRY1 activities that were not investigated here. Interestingly, multiple sequence alignment of cryptochromes from bryophyte, fern and seed plant species revealed sequence conservation of the VP motif related to CRY1 VP1 (Ponnu, 2020). Recent findings have demonstrated that the interaction between cryptochrome and COP1 from the moss *Physcomitrium patens* depends on the VP motif in the CCT domain of *P. patens* CRY1 (Kreiss et al., 2023). Apart from the conserved VP motif, the CCT domains of *P. patens* and Arabidopsis CRY1 show no major sequence conservation (Ponnu, 2020). This suggests that the VP motif might function as the primary signaling motif within the CCT domain of cryptochromes across at least 450 mio years of evolution. The VP‐mediated displacement of COP1 substrates by cryptochromes might therefore be an evolutionarily conserved mechanism among land plants. Moreover, the VP‐binding pocket in COP1‐WD is conserved between Arabidopsis and humans, with human COP1 (hCOP1) interacting with the VP motif in TRIB1 (Uljon et al., 2016). A distinct, but also somewhat analogous, competitive VP‐dependent protein–protein interaction has been reported for human COP1. The nuclear export of hCOP1 is promoted by an intramolecular interaction between the hCOP1‐WD domain and a VP‐containing regulatory site, named pseudosubstrate latch (PSL), within hCOP1 (Kung & Jura, 2019). This interaction depends on a VP motif in the PSL of hCOP1, which binds to the VP‐binding pocket of hCOP1‐WD. TRIB1 interacts via its VP motif with the VP‐binding pocket of hCOP1, thereby disrupting the interaction between PSL and hCOP1‐WD. As a result, the nuclear localization of hCOP1 is promoted (Kung & Jura, 2019). Though plant COP1, like mammalian COP1, shuttles between nucleus and cytoplasm (Arnim & Deng, 1994; Balcerowicz et al., 2017; Osterlund & Deng, 1998; Pacín et al., 2014), neither TRIB1 nor the PSL in COP1 exist (Kung & Jura, 2019). Thus, the molecular mechanism of nucleo‐cytoplasmic shuttling of Arabidopsis COP1 is likely distinct from that of human COP1.

Recently, it was shown that mammalian cryptochrome inhibits the activity of mammalian COP1. However, mammalian cryptochrome does not directly interact with mammalian COP1 but rather binds DET1 to disrupt the DET1‐COP1 interaction, thereby preventing the formation of a CRL4^COP1^ complex (Rizzini et al., 2019). Thus, the mechanisms of cryptochrome‐mediated inhibition of COP1 appears to be distinct between mammals and plants.

## MATERIALS AND METHODS

### Plant material

All Arabidopsis lines used in this study were in the Col‐0 accession. The *cry1‐304* and *cop1‐4* mutants were described before (McNellis et al., 1994; Mockler et al., 1999).

To generate transgenic CRY1, CRY1‐VP1^AA^, CRY1‐VP1^TRIB1‐VP*^, CRY1‐VP1^TRIB1‐VP:AA*^, CRY1‐VP1^TRIB1‐VP^ and CRY1‐VP1^TRIB1‐VP:AA^ lines, the corresponding constructs were generated as described in Methods S1 section and cloned into pFAST‐R06 (Shimada et al., 2010). They were then introduced into the *Agrobacterium tumefaciens* strain GV3101(pMP90) and subsequently transformed into *cry1‐304* by the floral‐dip method (Clough & Bent, 1998). For all constructs, independent transgenic lines homozygous for the respective transgene were generated. As the vector pFAST‐R06 contains OLE1:TagRFP cassette which causes accumulation of RFP in the oil bodies of the seeds (Shimada et al., 2010), transgenic lines generated and used in this study were selected based on the RFP fluorescence of their seeds using a fluorescence microscope (ZEISS Axio Zoom, Carl Zeiss AG, Germany).

### Growth conditions and phenotypic analyses

The LED light source for the blue light experiments was described previously (Laubinger et al., 2004). Transgenic plants expressing the CRY1 variants along with *cry1‐304* and Col‐0 were grown in blue light at a fluence rate between 2.5 and 10 μmol m^−2^ s^−1^ for hypocotyl measurements for 4–7 days. Fluence rates for each experiment are depicted in the corresponding figure legend. The hypocotyl length was analyzed as described previously (Laubinger et al., 2004).

For analysis of adult phenotypes, plants were grown in the greenhouse under long‐day conditions (16 h light, 8 h darkness) at approximately 40% humidity and a temperature cycle of 21°C during the day and 18°C during the night. They were grown at light intensities of approximately 100 μmol m^−2^ s^−1^ generated by Lumilux L36W/840 cool white fluorescent tubes (Osram, Munich, Germany).

The anthocyanin content of the seedlings was measured as described previously (Ponnu et al., 2019), except that 250 μL anthocyanin extraction buffer instead of 500 μL was used.

### Immunodetection of proteins

For detecting proteins, protein extraction was performed in 2× SDS buffer (125 mM Tris/HCl pH 6.8, 4% (w/v) SDS, 20% (v/v) glycerol, 1× protease inhibitor cocktail, 1 mM phenylmethylsulfonyl fluoride [PMSF]). Proteins were detected by α‐GFP‐HRP antibody (Miltenyi Biotec, Bergisch Gladbach, Germany), α‐COP1 (Balcerowicz et al., 2011) and α‐Tubulin (Sigma‐Aldrich, Munich, Germany). Signals were visualized in an ImageQuant™ LAS 4000 mini (GE Healthcare, Chicago, IL, USA).

### Protein–protein interaction methods

#### Yeast two‐ and three‐hybrid assays

Lex‐A‐based yeast two‐hybrid and Gal4‐based yeast three‐hybrid assays were performed as described by Ponnu et al. (2019). Transformed yeast cells were grown for 24 h in darkness before being either transferred to 50 μmol m^−2^ s^−1^ blue light or kept in darkness for 24 h.

#### Co‐localization analysis and FRET‐FLIM assays

For the co‐localization analyses and FRET‐FLIM assays of fluorescently tagged proteins, leek epidermal cells were transiently transformed with plasmids by particle bombardment as described by Ponnu et al. (2019). Fluorescent proteins were detected using an SP8 confocal laser scanning microscope (Leica Microsystems, Wetzlar, Germany). FRET‐FLIM analyses were conducted as described by Ponnu et al. (2019).

#### Co‐immunoprecipitation

For *in vivo* co‐immunoprecipitations, seedlings were homogenized in extraction buffer (50 mM Tris/HCl pH 7.5, 150 mM NaCl, 500 μM EDTA pH 8.0, 1% (v/v) Triton‐X‐100, 1% (v/v) protease inhibitor cocktail (Sigma Aldrich, Munich, Germany)). After centrifugation, 1 mg of total protein lysate was subjected to co‐immunoprecipitation using the magnetic μMACS™ GFP isolation kit (Miltenyi Biotec, Bergisch Gladbach, Germany) for GFP‐tagged bait proteins and subsequently washed according to the manufacturer's instructions. Proteins were detected by western blot using antibodies against GFP, COP1 and Tubulin as described above.

#### For luciferase complementation (LCI) imaging assay

Luciferase complementation imaging (LCI) was performed as described in (Kreiss et al., 2023). In short, nLUC and cLUC fusion proteins were expressed in transfected *N. benthamiana* leaves and the luminescence was imaged with a CCD‐based camera (ImageQuantTM LAS 4000) mini‐imaging system (GE Healthcare, Piscataway, USA). The signals were quantified by Image J 1.53 software (Wayne Rasband, NIH Image Software, National Institutes of Health; Bethesda, USA). Afterwards, the luminescence, GFP and RFP signals were quantified using the Infinite RM200 plate reader (Tecan, Männedorf, Switzerland, Figure S2A). For this, leaves were washed with water to remove external luciferin solution. For each infiltrated area, five to eight leaf discs (approx. 5 mm diameter) were punched out and placed with the adaxial side facing top in a LUMITRAC 600 Micro Plate (Greiner, Kremsmünster, Austria) containing 200 μL ddH_2_O. Then, 20 μL D‐Luciferin potassium salt (Synchem UG & Co. KG (Felsberg/Altenburg, Germany)) was added. The luminescence was detected by the plate reader for 1000 ms. The tagRFP (Schwenk et al., 2021) was excited at 555 nm and the emission was detected at 584 nm. GFP was excited at 483 nm and its emission was detected at 535 nm. For GFP and RFP detection, the gain was set to optimal and the number of flashes was adjusted to 40. Quantification of the plate reader data was carried out as follows: (1) For each infiltrated area (area 1–4), the 5–8 values that correspond to the number of leaf discs were averaged for the GFP, RFP and luminescence values. In addition, the mean of the blank sample (leaf disks of an uninfiltrated area) was determined for each signal. (2) The signal of the blank was subtracted from the area values of the respective signals of the infiltrated areas to account for the background signal, to get the ‘adjusted value’. (3) Next, the adjusted value for area 1/2/3/4 was divided by the adjusted value for area 1. This calculation was repeated for all tested leaves and all signals. The normalized values were plotted.

#### Mammalian three‐hybrid assay

All plasmids were constructed using AQUA cloning (Beyer et al., 2015). All plasmid information used in this work in animal cells is documented and curated in the GMOCU platform (DOI: https://doi.org/10.1002/adbi.202300529). Approximately 50.000 CHO‐K1, DSMZ ACC 110 cells per well were seeded onto 24‐well plates in a total volume of 500 μL Ham's F12 Medium (PAN Biotech) supplemented with 125 U/mL penicillin, 125 U/mL streptomycin and 10% FBS (PAN Biotech, cat. no. P30‐3602). The cells were then incubated for ~24 h at 37°C in a 5% CO_2_ atmosphere. Consecutively, 750 ng DNA‐mix was transfected in equimolar plasmid amounts (w/w) using polyethyl‐enimine (Polysciences Inc. Europe). After 4 h, the medium was exchanged. 24 h after the transfection the medium was exchanged again prior to 24 h of darkness or illumination with blue light using custom‐made LED arrays. The reporter SEAP was quantified in the cell culture medium as described before (Müller et al., 2013). The normalization element secreted NanoLuciferase (secNLuc) was quantified by taking 80 μL of cell culture medium, adding coelenterazine (Carl Roth GmbH) and measuring luminescence for 20 min (TriStar2 LB 942 multimode plate reader, Berthold Technologies).

### Cloning of constructs

Plasmid construction is described in detail in Methods S1.

### Statistical analyses

For statistical analysis and data visualization GraphPad Prism (GraphPad Prism version 10.2.3 for Windows, GraphPad Software, Boston, Massachusetts USA, www.graphpad.com) was used. Upper and lower hinges of the boxplots correspond to the minimum and maximum value. Each individual replicate is plotted as a dot. The median is represented by a horizontal line within the box. The mean is represented by a ‘+’. To determine statistically significant differences between means, one‐ or two‐way analysis of variance (anova) was followed by a Tukey's honestly significant difference (HSD) test.

## Author Contributions

UH and LT designed experiments and wrote the manuscript. LT performed most of the experiments. All M2H and M3H experiments were designed and performed by FK and MDZ. JP generated the GFP‐CRY1 and GFP‐CRY1‐VP1^AA^ transgenic lines. KK and LT analyzed the hypocotyl phenotype of the CRY1‐VP1^AA^ transgenic lines. PY and LT analyzed the hypocotyl phenotype of the chimeric CRY1‐TRIB1 lines. PL and LT analyzed the COP1‐CRY1 chimera interaction in yeast. UH, LT, FK, MDZ, KK, PY and PL analyzed the data. All authors discussed the results and read and commented on the manuscript.

## Conflict of Interest Statement

All authors declare that they have no competing interests.

## Supporting information


**Figure S1.** Conservation of cryptochrome 1‐VP motifs in representative angiosperms.
**Figure S2.** Quantification of Luciferase complementation imaging (LCI) assay showing the interaction of COP1 and CRY1.
**Figure S3.** The VP1 motif is essential for the interaction of CCT1 with COP1.
**Figure S4.** COP1 recruits PAP2 into co‐localizing nuclear bodies.
**Figure S5.** Quantification of luciferase complementation imaging assay showing the interaction of COP1 and PAP2 in the presence of GFP‐NLS‐GUS, GFP‐CRY1 or GFP‐CRY1‐VP1^AA^.
**Figure S6.** The effect of CCT1 coexpression on the COP1‐PAP2 interaction.
**Figure S7.** Quantification of luciferase complementation imaging assay showing the interaction of COP1 and HY5 in the presence of GFP‐NLS‐GUS, GFP‐CRY1 or GFP‐CRY1‐VP1^AA^.
**Figure S8.** CRY1 interacts with COP1 via its VP1 motif in the mammalian two‐hybrid system.
**Figure S9.** CRY1‐TRIB1 chimeras interact with COP1.
**Figure S10.** Immunoblot analysis of transgenic *cry1‐304* seedlings expressing GFP‐CRY1, GFP‐CRY1‐VP1^AA^, GFP‐CRY1‐VP1^TRIB1‐VP^*, GFPCRY1‐VP1^TRIB1‐VP:AA*^, GFP‐CRY1‐VP1^TRIB1‐VP^ or GFP‐CRY1‐VP1^TRIB1‐VP:AA^ under the control of the *35S* promoter.
**Figure S11.** None of the tested GFP‐CRY1‐VP1^TRIB1‐VP:AA^ *cry1‐304* lines complemented the *cry1‐304* mutant phenotype.
**Methods S1.** Cloning of constructs used in this study.
**Table S1.** List of primers used in this study.
**Table S2.** List of materials used in this study.

## Data Availability

The data that supports the findings of this study are available in the supplementary material of this article.
