# Supplementary material for: Cryptochrome 1 promotes photomorphogenesis in Arabidopsis by displacing substrates from the COP1 ubiquitin ligase
Source: Plant J. 2025 Mar 7;121(5):e70071. doi: 10.1111/tpj.70071 (PMC11886768; doi:10.1111/tpj.70071)
Supplement: Supplementary file 1 — Figure S1. Conservation of cryptochrome 1‐VP motifs in representative angiosperms. Figure S2. Quantification of Luciferase complementation imaging (LCI) assay showing the interaction of COP1 and CRY1. Figure S3. The VP1 motif is essential for the interaction of CCT1 with COP1. Figure S4. COP1 recruits PAP2 into co‐localizing nuclear bodies. Figure S5. Quantification of luciferase complementation imaging assay showing the interaction of COP1 and PAP2 in the presence of GFP‐NLS‐GUS, GFP‐CRY1 or GFP‐CRY1‐VP1AA. Figure S6. The effect of CCT1 coexpression on the COP1‐PAP2 interaction. Figure S7. Quantification of luciferase complementation imaging assay showing the interaction of COP1 and HY5 in the presence of GFP‐NLS‐GUS, GFP‐CRY1 or GFP‐CRY1‐VP1AA. Figure S8. CRY1 interacts with COP1 via its VP1 motif in the mammalian two‐hybrid system. Figure S9. CRY1‐TRIB1 chimeras interact with COP1. Figure S10. Immunoblot analysis of transgenic cry1‐304 seedlings expressing GFP‐CRY1, GFP‐CRY1‐VP1AA, GFP‐CRY1‐VP1TRIB1‐VP*, GFPCRY1‐VP1TRIB1‐VP:AA*, GFP‐CRY1‐VP1TRIB1‐VP or GFP‐CRY1‐VP1TRIB1‐VP:AA under the control of the 35S promoter. Figure S11. None of the tested GFP‐CRY1‐VP1TRIB1‐VP:AA cry1‐304 lines complemented the cry1‐304 mutant phenotype. Methods S1. Cloning of constructs used in this study. Table S1. List of primers used in this study. Table S2. List of materials used in this study. [file TPJ-121-0-s001.pdf]

Supporting Information

Article title: Cryptochrome 1 promotes photomorphogenesis in Arabidopsis by displacing substrates from the COP1 ubiquitin ligase

Authors: Laura Trimborn, Franziska Kuttig, Jathish Ponnu, Pengxin Yu, Kris R. Korsching, Patrick Lederer, Uriel Urquiza-García, Matias D. Zurbriggen, Ute Hoecker [*]

*Corresponding author: Ute Hoecker; Email: hoeckeru@uni-koeln.de

The following Supporting Information is available for this article:

Fig. S1: Conservation of cryptochrome 1 -VP motifs in representative angiosperms.

Fig. S2: Quantification of Luciferase complementation imaging (LCI) assay showing the interaction of COP1 and CRY1.

Fig. S3: The VP1 motif is essential for the interaction of CCT1 with COP1.

Fig. S4: COP1 recruits PAP2 into co-localizing nuclear bodies.

Fig. S5: Quantification of luciferase complementation imaging assay showing the interaction of COP1 and PAP2 in the presence of GFP-NLS-GUS, GFP-CRY1 or GFP-CRY1-VP1^{AA}.

Fig. S6: GFP-NLS-GUS-CCT1, but not GFP-NLS-CCT1, inhibits COP1-PAP2 interaction.

Fig. S7: Quantification of luciferase complementation imaging assay showing the interaction of COP1 and HY5 in the presence of GFP-NLS-GUS, GFP-CRY1 or GFP-CRY1-VP1^{AA}.

Fig. S8: CRY1 interacts with COP1 via its VP1 motif in the mammalian two-hybrid system.

Fig. S9: CRY1-TRIB1 chimeras interact with COP1.

Fig. S10: Immunoblot analysis of transgenic *cry1-304* seedlings expressing GFP-CRY1, GFP-CRY1-VP1^{AA}, GFP-CRY1-VP1$^{TRIB1\text{-}VP}$*, GFPCRY1-VP1$^{TRIB1\text{-}VP:AA*,}$ GFP-CRY1-VP1$^{TRIB1\text{-}VP}$ or GFP-CRY1-VP1$^{TRIB1\text{-}VP:AA}$ under the control of the *35S* promoter.

Fig. S11: None of the tested GFP-CRY1-VP1$^{TRIB1\text{-}VP:AA}$ *cry1-304* lines complemented the *cry1-304* mutant phenotype.

Methods S1: Cloning of constructs used in this study.

Table S1: List of primers used in this study.

Table S2: List of materials used in this study.

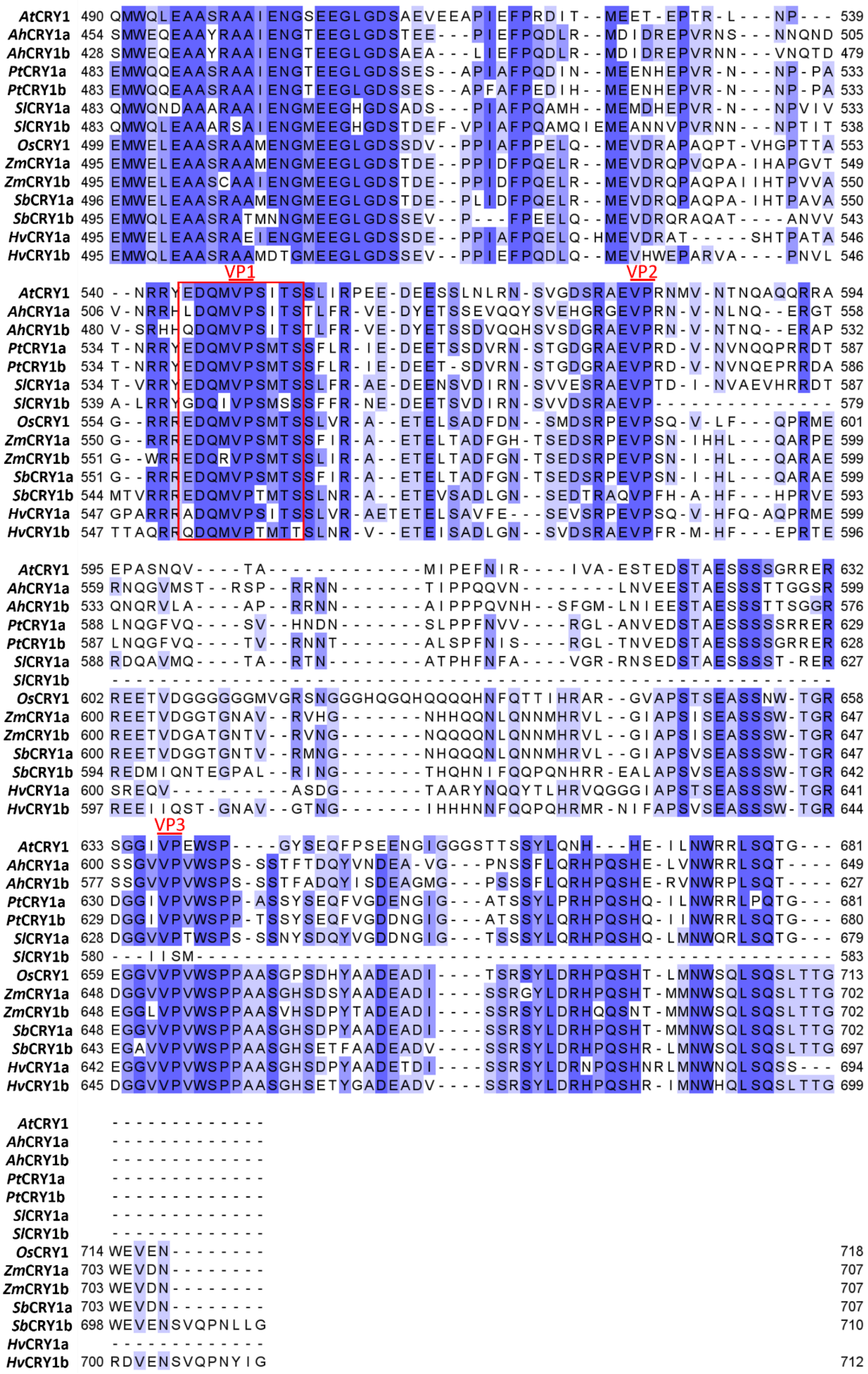
AtCRY1 490 QMWQLEAASRAAIENGSEEGLGDSAEVEEAPIEFPRDIT--MEET-EPTR-L---NP--- 539
AhCRY1a 454 SMWEQEAAYRAAIENGTEEGLGDSTEE---PIEFPQDLR--MDIDREPVRNS---NNQND 505
AhCRY1b 428 SMWEQEAAYRAAIENGTEEGLGDSAEA---LIEFPQDLR--MDIDREPVRNN---VNQTD 479
PtCRY1a 483 EMWQQEAASRAAIENGTEEGLGDSSES--APIAFPQDIN--MEENHEPVR-N---NP-PA 533
PtCRY1b 483 EMWQQEAASRAAIENGTEEGLGDSSES--APFAFPEDIH--MEENHEPVR-N---NP-PA 533
SlCRY1a 483 QMWQNDAAARAAIENGMEEGHGDSADS---PIAFPQAMH--MEMDHEPVR-N---NPVIV 533
SlCRY1b 483 QMWQLEAAARSAIENGMEEGHGDSTDEF-VPIAFPQAMQIEMEANNVPVRNN---NPTIT 538
OsCRY1 499 EMWELEAASRAAMENGMEEGLGDSSDV--PPIAFPPELQ--MEVDRAPAQPT-VHGPTTA 553
ZmCRY1a 495 EMWELEAASRAAMENGMEEGLGDSTDE--PPIDFPQELR--MEVDRQPVQPA-IHAPGVT 549
ZmCRY1b 495 EMWELEAASCAAIENGMEEGLGDSTDE--PPIDFPQELR--MEVDRQPAQPAIIHTPVVA 550
SbCRY1a 496 EMWELEAASRAAMENGMEEGLGDSTDE--PLIDFPQELR--MEVDRQPAQPA-IHTPAVA 550
SbCRY1b 495 EMWQLEAASRATMNNGMEEGLGDSSEV--P---FPEELQ--MEVDRQRAQAT----ANVV 543
HvCRY1a 495 EMWELEAASRAEIENGMEEGLGDSSDE--PPIAFPQELQ-HMEVDRAT-----SHTPATA 546
HvCRY1b 495 EMWQLEAASRAAMDTGMEEGLGDSSEV--PPIEFPQDLQ--MEVHWEPARVA----PNVL 546
VP1
VP2
AtCRY1 540 --NRRYEDQMVPSITSSLIRPEE-DEESSLNLRN-SVGDSRAEVPRNMV-NTNQAQQRRA 594
AhCRY1a 506 V-NRRHLDQMVPSITSTLFR-VE-DYETSSEVQQYSVEHGRGEVPRN-V-NLNQ--ERGT 558
AhCRY1b 480 V-SRHHQDQMVPSITSTLFR-VE-DYETSSDVQQHSVSDGRAEVPRN-V-NTNQ--ERAP 532
PtCRY1a 534 T-NRRYEDQMVPSMTSSFLR-IE-DEETSSDVRN-STGDGRAEVPRD-V-NVNQQPRRDT 587
PtCRY1b 534 T-NRRYEDQMVPSMTSSFLR-IE-DEET-SDVRN-STGDGRAEVPRD-V-NVNQEPRRDA 586
SlCRY1a 534 T-VRRYEDQMVPSMTSSLFR-AE-DEENSVDIRN-SVVESRAEVPTD-I-NVAEVHRRDT 587
SlCRY1b 539 A-LRRYGDQIVPSMSSSFFR-NE-DEETSVDIRN-SVVDSRAEVP-------------- 579
OsCRY1 554 G--RRREDQMVPSMTSSLVR-A--ETELSADFDN--SMDSRPEVPSQ-V-LF---QPRME 601
ZmCRY1a 550 G--RRREDQMVPSMTSSFIR-A--ETELTADFGH-TSEDSRPEVPSN-IHHL---QARPE 599
ZmCRY1b 551 G--WRREDQRVPSMTSSLIR-A--ETELTADFGN-TSEDSRPEVPSN-I-HL---QARAE 599
SbCRY1a 551 G--RRREDQMVPSMTSSFIR-A--ETELTADFGN-TSEDSRPEVPSN-I-HL---QARAE 599
SbCRY1b 544 MTVRRREDQMVPTMTSSLNR-A--ETEVSADLGN--SEDTRAQVPFH-A-HF---HPRVE 593
HvCRY1a 547 GPARRRADQMVPSITSSLVR-AETETELSAVFE---SEVSRPEVPSQ-V-HFQ-AQPRME 599
HvCRY1b 547 TTAQRRQDQMVPTMTTSLNR-V--ETEISADLGN--SVDSRAEVPFR-M-HF---EPRTE 596
AtCRY1 595 EPASNQV----TA-------------MIPEFNIR----IVA-ESTEDSTAESSSSGRRER 632
AhCRY1a 559 RNQGVMST--RSP--RRNN-------TIPPQQVN--------LNVEESTAESSSTTGGSR 599
AhCRY1b 533 QNQRVLA----AP--RRNN-------AIPPPQVNH--SFGM-LNIEESTAESSSTTSGGR 576
PtCRY1a 588 LNQGFVQ----SV--HNDN-------SLPPFNVV----RGL-ANVEDSTAESSSSSRRER 629
PtCRY1b 587 LNQGFVQ----TV--RNNT-------ALSPFNIS----RGL-TNVEDSTAESSSSGRRER 628
SlCRY1a 588 RDQAVMQ----TA--RTN--------ATPHFNFA----VGR-RNSEDSTAESSSST-RER 627
SlCRY1b ---
OsCRY1 602 REETVDGGGGGGMVGRSNGGGHQGQHQQQQHNFQTTIHRAR--GVAPSTSEASSNW-TGR 658
ZmCRY1a 600 REETVDGGTGNAV--RVHG-------NHHQQNLQNNMHRVL--GIAPSISEASSSW-TGR 647
ZmCRY1b 600 REETVDGATGNTV--RVNG-------NQQQQNLQNNMHRVL--GIAPSISEASSSW-TGR 647
SbCRY1a 600 REETVDGGTGNTV--RMNG-------NHQQQNLQNNMHRVL--GIAPSVSEASSSW-TGR 647
SbCRY1b 594 REDMIQNTEGPAL--RING-------THQHNIFQQPQNHRR-EALAPSVSEASSSW-TGR 642
HvCRY1a 600 SREQV----------ASDG-------TAARYNQQYTLHRVQGGGIAPSTSEASSSW-TGR 641
HvCRY1b 597 REEIIQST-GNAV--GTNG-------IHHHNNFQQPQHRMR-NIFAPSVSEASSSW-TGR 644
VP3
AtCRY1 633 SGGIVPEWSP----GYSEQFPSEENGIGGGSTTSSYLQNH---HE-ILNWRRLSQTG--- 681
AhCRY1a 600 SSGVVPVWSPS-SSTFTDQYVNDEA-VG---PNSSFLQRHPQSHE-LVNWRRLSQT---- 649
AhCRY1b 577 SSGVVPVWSPS-SSTFADQYISDEAGMG---PSSSFLQRHPQSHE-RVNWRPLSQT---- 627
PtCRY1a 630 DGGIVPVWSPP-ASSYSEQFVGDENGIG---ATSSYLPRHPQSHQ-ILNWRRLPQTG--- 681
PtCRY1b 629 DGGIVPVWSPP-TSSYSEQFVGDDNGIG---ATSSYLQRHPQSHQ-IINWRRLSQTG--- 680
SlCRY1a 628 DGGVVPTWSPS-SSNYSDQYVGDDNGIG---TSSSYLQRHPQSHQ-LMNWQRLSQTG--- 679
SlCRY1b 580 ---IISM-- 583
OsCRY1 659 EGGVVPVWSPPAASGPSDHYAADEADI----TSRSYLDRHPQSHT-LMNWSQLSQSLTTG 713
ZmCRY1a 648 DGGVVPVWSPPAASGHSDSYAADEADI----SSRGYLDRHPQSHT-MMNWSQLSQSLTTG 702
ZmCRY1b 648 EGGLVPVWSPPAASVHSDPYTADEADI----SSRSYLDRHQQSNT-MMNWSQLSQSLTTG 702
SbCRY1a 648 EGGVVPVWSPPAASGHSDPYAADEADI----SSRSYLDRHPQSHT-MMNWSQLSQSLTTG 702
SbCRY1b 643 EGAVVPVWSPPAASGHSETFAADEADV----SSRSYLDRHPQSHR-LMNWSQLSQSLTTG 697
HvCRY1a 642 EGGVVPVWSPPAASGHSDPYAADETDI----SSRSYLDRNPQSHNRLMNWNQLSQSS--- 694
HvCRY1b 645 DGGVVPVWSPPAASGHSETYGADEADV----SSRSYLDRHPQSHR-IMNWHQLSQSLTTG 699
AtCRY1 -------------
AhCRY1a -------------
AhCRY1b -------------
PtCRY1a -------------
PtCRY1b -------------
SlCRY1a -------------
SlCRY1b -------------
OsCRY1 714 WEVEN-------- 718
ZmCRY1a 703 WEVDN-------- 707
ZmCRY1b 703 WEVDN-------- 707
SbCRY1a 703 WEVDN-------- 707
SbCRY1b 698 WEVENSVQPNLLG 710
HvCRY1a -------------
HvCRY1b 700 RDVENSVQPNYIG 712

Fig. S1: Conservation of cryptochrome 1-VP motifs in representative angiosperms.
Amino acid alignment of the CCT domains. The multiple sequence alignment was performed using Jalview (Mafft Default Settings). The framed amino acids are the core VP motif which includes VP1.
VP1, VP2 and VP3 are indicated above the amino acid sequence.
The intensity of blue color represents the percentage identity.
At, Arabidopsis thaliana; Ah, Amaranthus hypochondriacus; Pt, Populus trichocarpa; Sl, Solanum lycopersicum; Os, Oryza sativa; Zm,Zea mays; Sb, Sorghum bicolor; Hv, Hordeum vulgare.

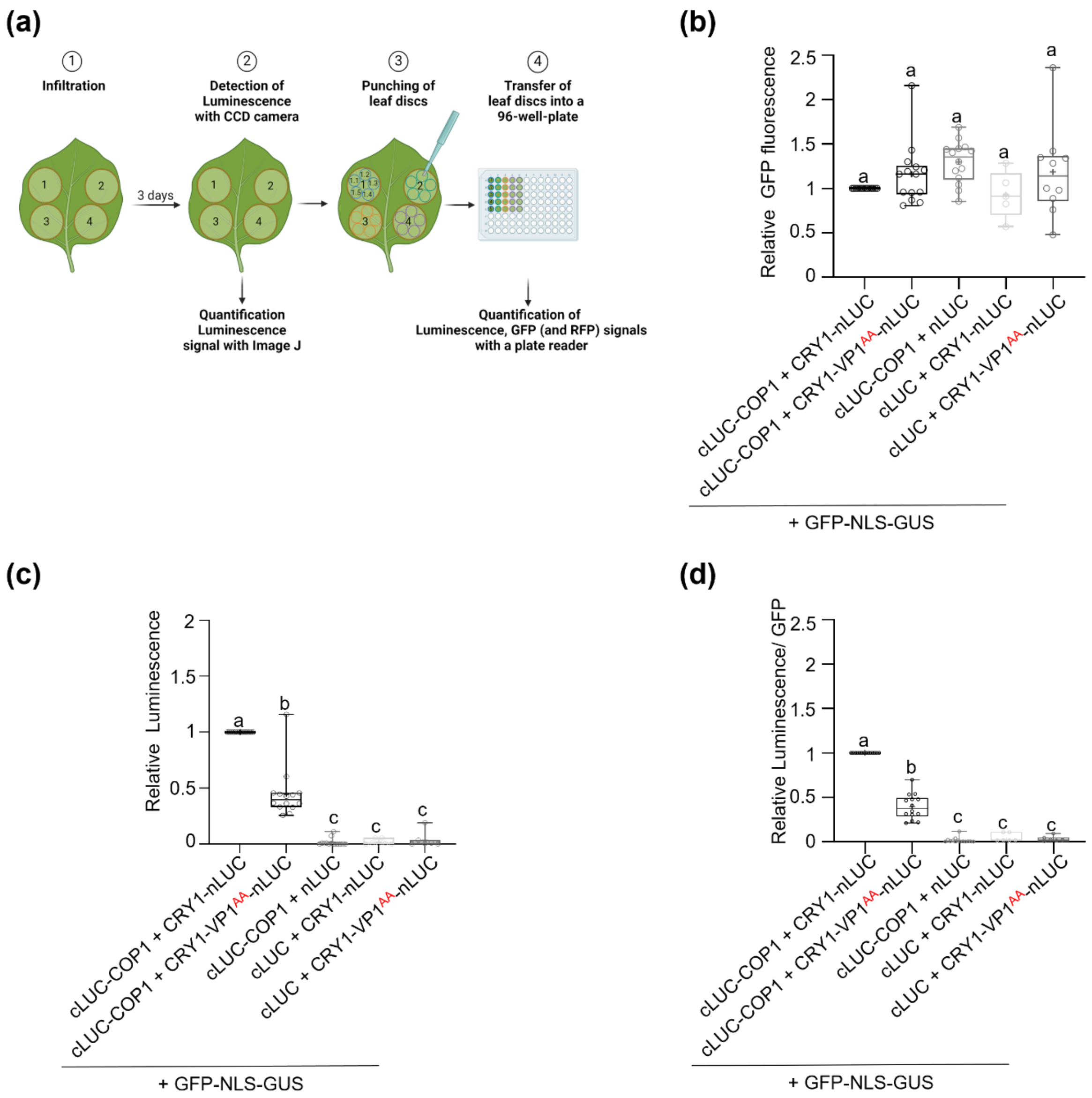

Fig. S2: Quantification of Luciferase complementation imaging (LCI) assay showing the interaction of COP1 and CRY1. **(a)** Analysis of LCI assay. (1) *N. benthamiana* leaf was infiltrated with *Agrobacterium tumefaciens* cell suspension at four distinct areas (area 1, 2, 3 and 4). (2) Three days after infiltration luminescence was imaged with a CCD-based camera and the signals were quantified by Image J. (3) Then, of the same leaf, for each infiltrated area, approximately 5 leaf discs were punched out. (4) Leaf discs were placed in a 96-well plate and the luminescence signal, the GFP and, if present, RFP emissions were detected by a plate reader. Created in BioRender. Hoecker, U. (2023) BioRender.com/a87k930. **(b-d)** COP1 was fused to cLUC, and CRY1 or CRY1 with VP1 mutated to Ala residues (CRY1-VP1^{AA}) were fused to nLUC. As expression control, GFP-NLS-GUS plasmid was coexpressed. **(b)** GFP signals and **(c)** Luminescence signals of leaves analyzed in Fig. 1C, were detected using an Infinite RM200 plate reader. **(d)** Luminescence was normalized to the GFP signal. The box plots show the respective signals for n = 14 leaves measured, except for the negative controls cLUC + CRY1-nLUC and cLUC + CRY1-VP1^{AA}-nLUC, which shows the signal of n = 5-10 leaves each; relative to the signal of cLUC-COP1 + CRY1-nLUC. Statistical differences were estimated using one-way ANOVA followed by Tukey's multiple comparison. Different letters indicate statistically significant differences ($p \leq 0.05$).

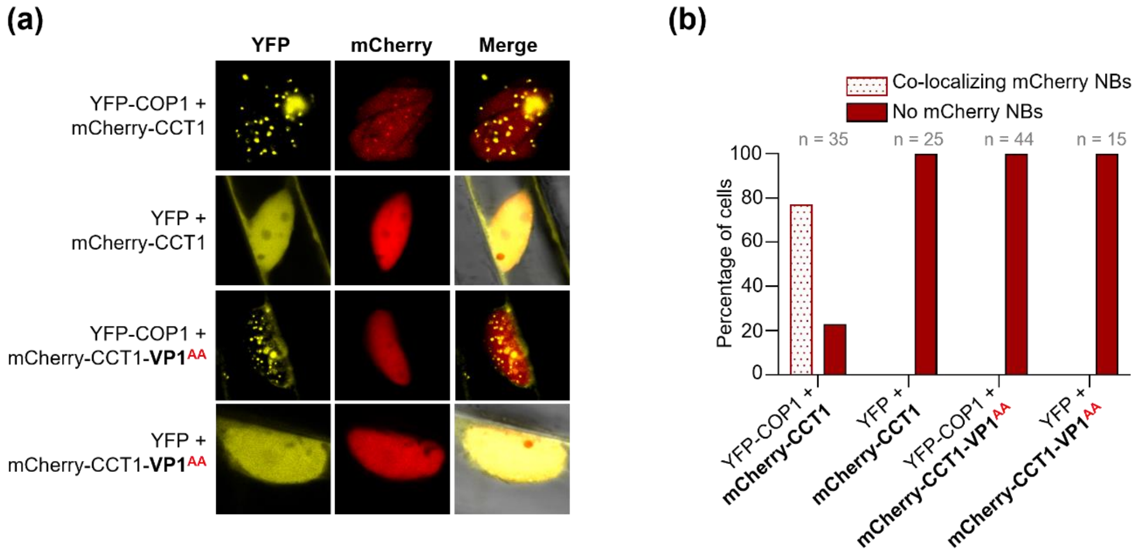

Fig. S3: The VP1 motif is essential for the interaction of CCT1 with COP1. (a) Representative confocal images of leek epidermal cells co-expressing mCherry-CCT1 or mCherry-CCT1-VP1^{AA} and YFP-COP1 or YFP after particle bombardment. **(b)** Percentage of cells that formed mCherry nuclear bodies (NBs) when indicated fusion proteins were co-expressed in leek epidermal cells. The number of cells analyzed is indicated above the bar.

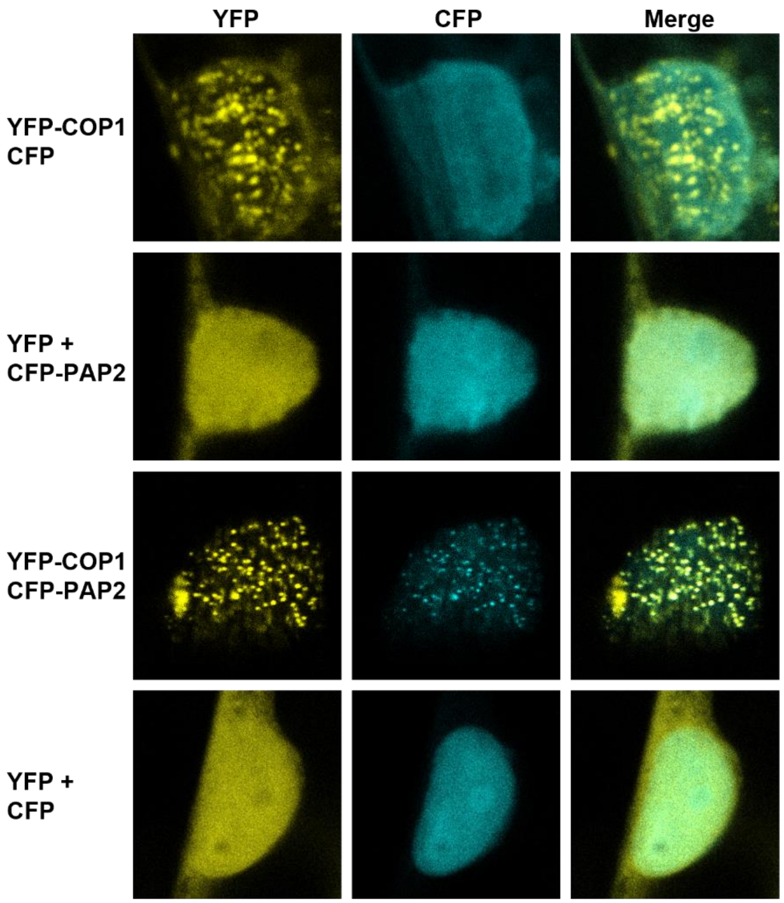

Fig. S4: COP1 recruits PAP2 into co-localizing nuclear bodies. Representative confocal images of leek epidermal cells coexpressing the indicated fusion proteins after particle bombardment. The representative confocal images for each channel were merged to show co-localization. The experiment was repeated twice.

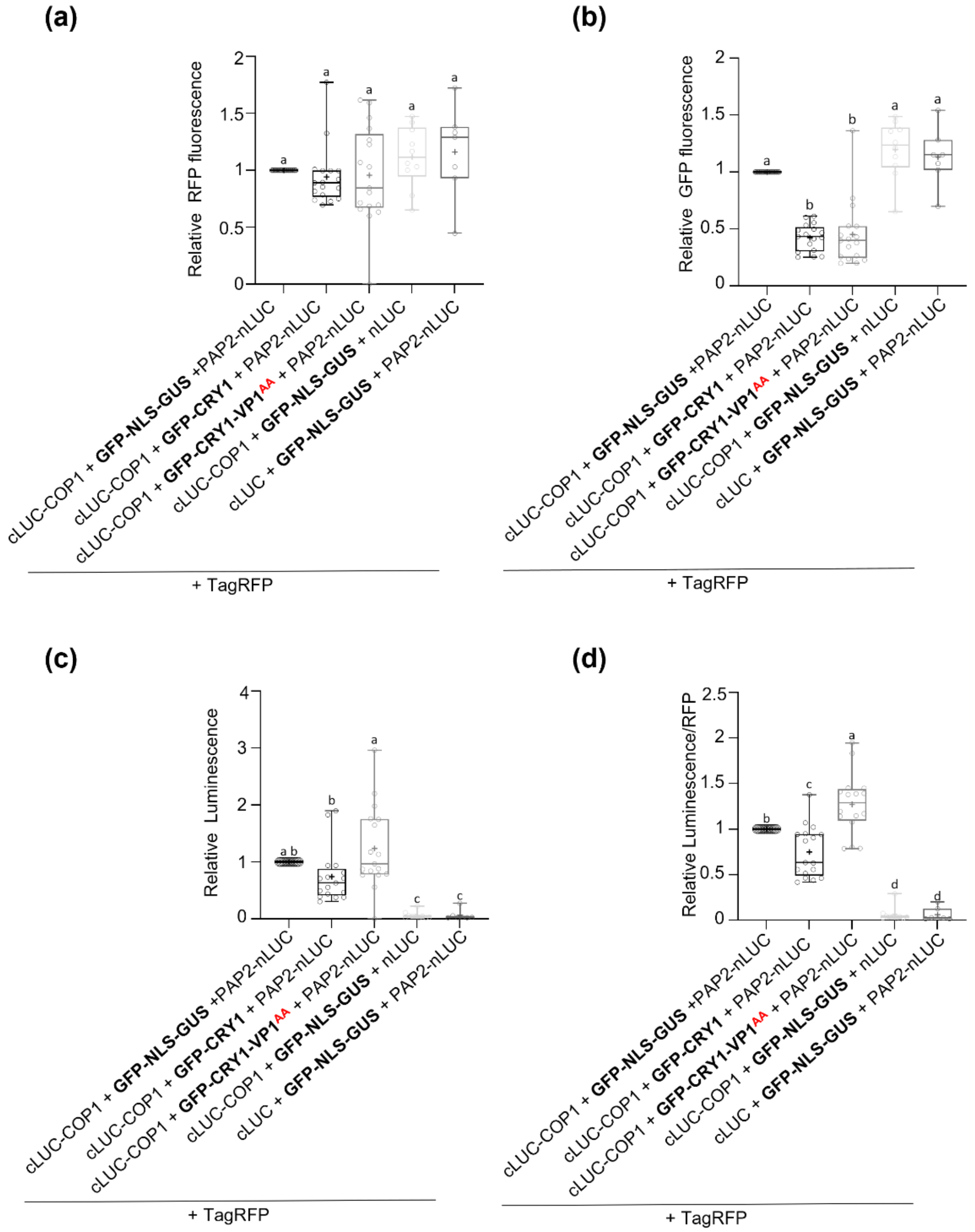

Fig. S5: Quantification of luciferase complementation imaging assay showing the interaction of COP1 and PAP2 in the presence of GFP-NLS-GUS, GFP-CRY1 or GFP-CRY1-VP1^{AA}. COP1 was fused to cLUC and PAP2 was fused to nLUC. As expression control, TagRFP was coexpressed. **(a)** RFP signals **(b)** GFP signals and **(c)** Luminescence signals of leaves analyzed in Figure 3D, were detected using an Infinite RM200 plate reader. **(d)** Luminescence was normalized to the RFP signal. The box plots show the respective signals for n = 16 leaves measured relative to the signal of cLUC-COP1 + GFP-NLS-GUS + PAP2- nLUC. , except for the negative controls, which shows the signal of n = 10 (cLUC- COP1 + GFP-NLS-GUS + nLUC) or 6 (cLUC + GFP-NLS-GUS + PAP2-nLUC) leaves; Hinges, Whiskers, median and mean are represented as explained above. Statistical differences were estimated using one-way ANOVA followed by Tukey's multiple comparison. Different letters indicate statistically significant differences ($p \leqslant 0.05$).

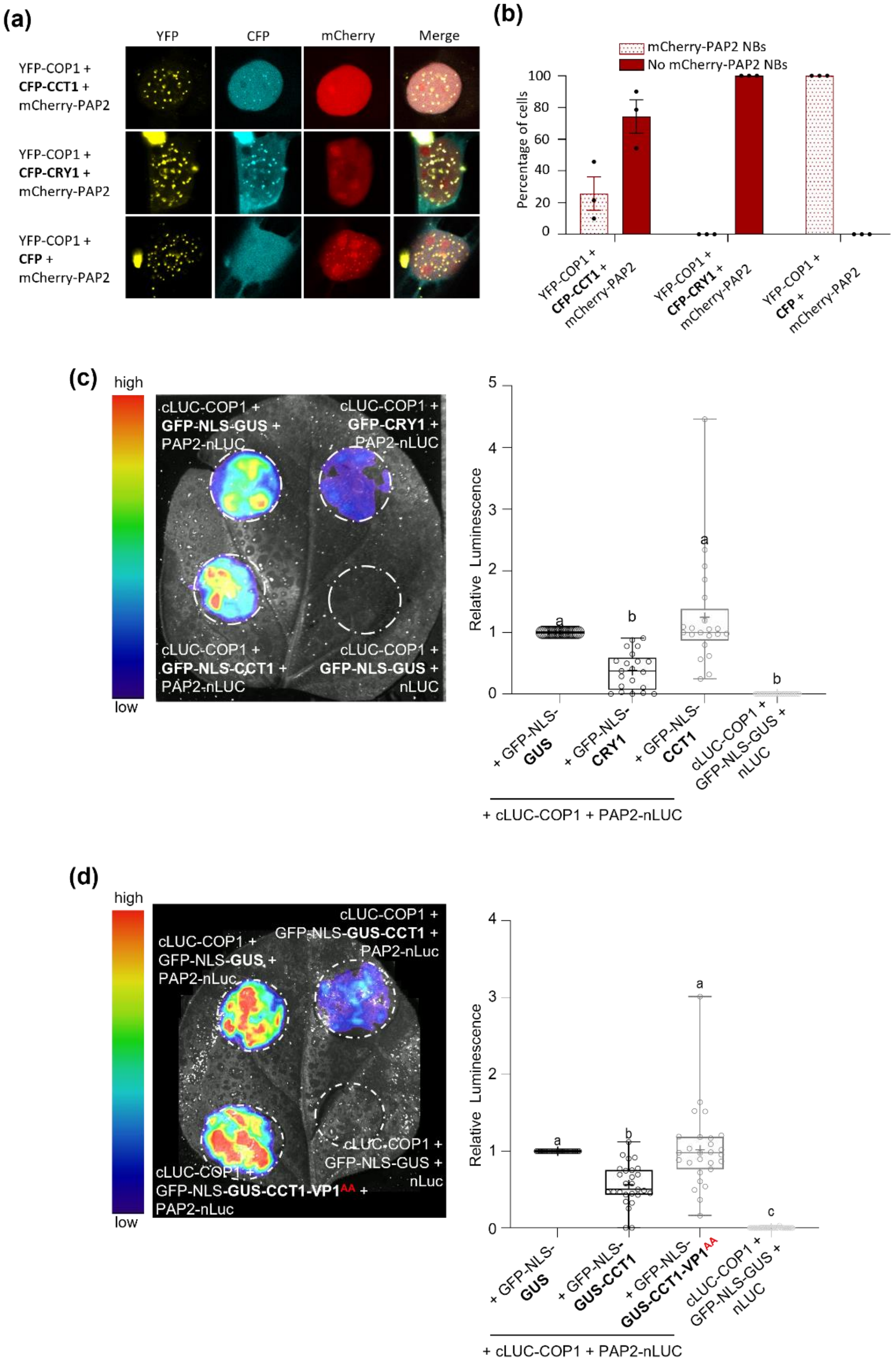

Fig. S6: The effect of CCT1 coexpression on the COP1-PAP2 interaction(a) Representative confocal images of leek epidermal cells co-expressing the indicated fusion proteins after particle bombardment. **(b)** Percentage of cells that formed mCherry-PAP2 nuclear bodies (NBs) co-localizing with YFP-COP1 when indicated fusion proteins were co-expressed in leek epidermal cells. The experiment was repeated three times, with 25 cells being evaluated for each combination in each experiment. Bars show the mean

± SEM. Dots represent the mean value of the independent experiments. **(c, d)** LCI assay showing the interaction of COP1 and PAP2 in presence of **(c)** GFP-NLS-GUS, GFP-CRY1, or GFP-NLS-CCT1 or **(d)** GFP-NLS-GUS, GFP-NLS-GUS-CCT1 or GFP-NLS-GUS-CCT1-VP1^{AA}. COP1 was fused to cLUC and PAP2 to nLUC. (Left) Image of luminescence in a representative transfected *N. benthamiana* leaf. (Right) Relative luminescence signal in transfected leaves as measured using a CCD camera. The box plots show the relative luminescence signal for (c) n = 21 or (d) n = 26 leaves. Hinges, Whiskers, median and mean are represented as explained above. Statistical differences were estimated using one-way ANOVA followed by Tukey's multiple comparison. Different letters indicate statistically significant differences ($p \leqslant 0.05$). The experiments were repeated twice with similar results.

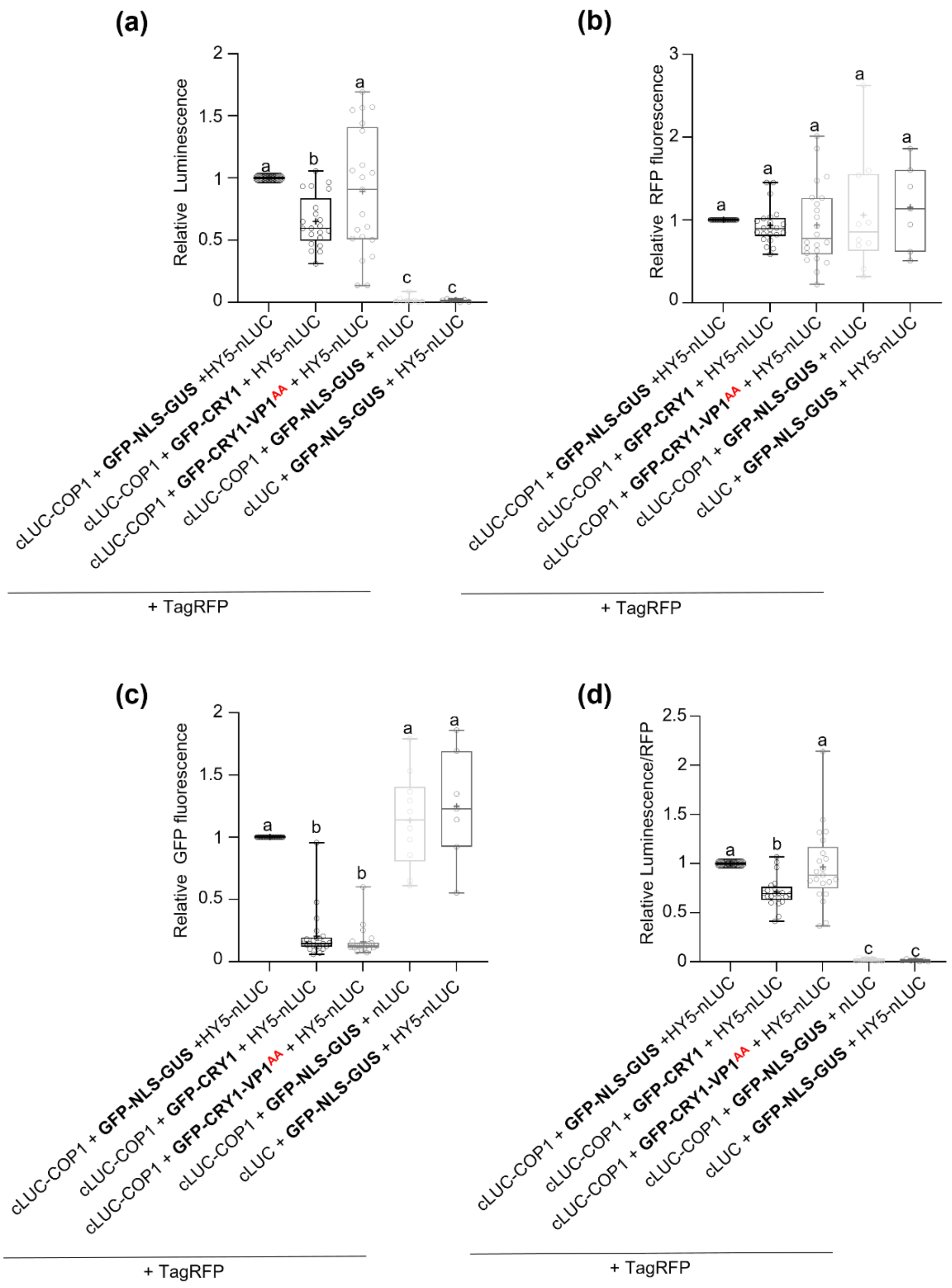

Fig. S7: Quantification of luciferase complementation imaging assay showing the interaction of COP1 and HY5 in the presence of GFP-NLS-GUS, GFP-CRY1 or GFP-CRY1-VP1^{AA}. COP1 was fused to cLUC and HY5 was fused to nLUC. As expression control, TagRFP was coexpressed. **(a)** Luminescence signal, **(b)** RFP signal and **(c)** GFP signal of leaves analyzed in Figure 4A were detected using an Infinite RM200 plate reader. **(d)** Tag-RFP-normalized luminescence (luminescence/TagRFP) . The box plots show the respective signals for n = 22 leaves measured relative to the signal of cLUC-COP1 + GFP-NLS-GUS + HY5-nLUC. Hinges, Whiskers, median and mean are represented as explained above. Statistical differences were estimated using

one-way ANOVA followed by Tukey’s multiple comparison. Different letters indicate statistically significant differences ($p \leqslant 0.05$).

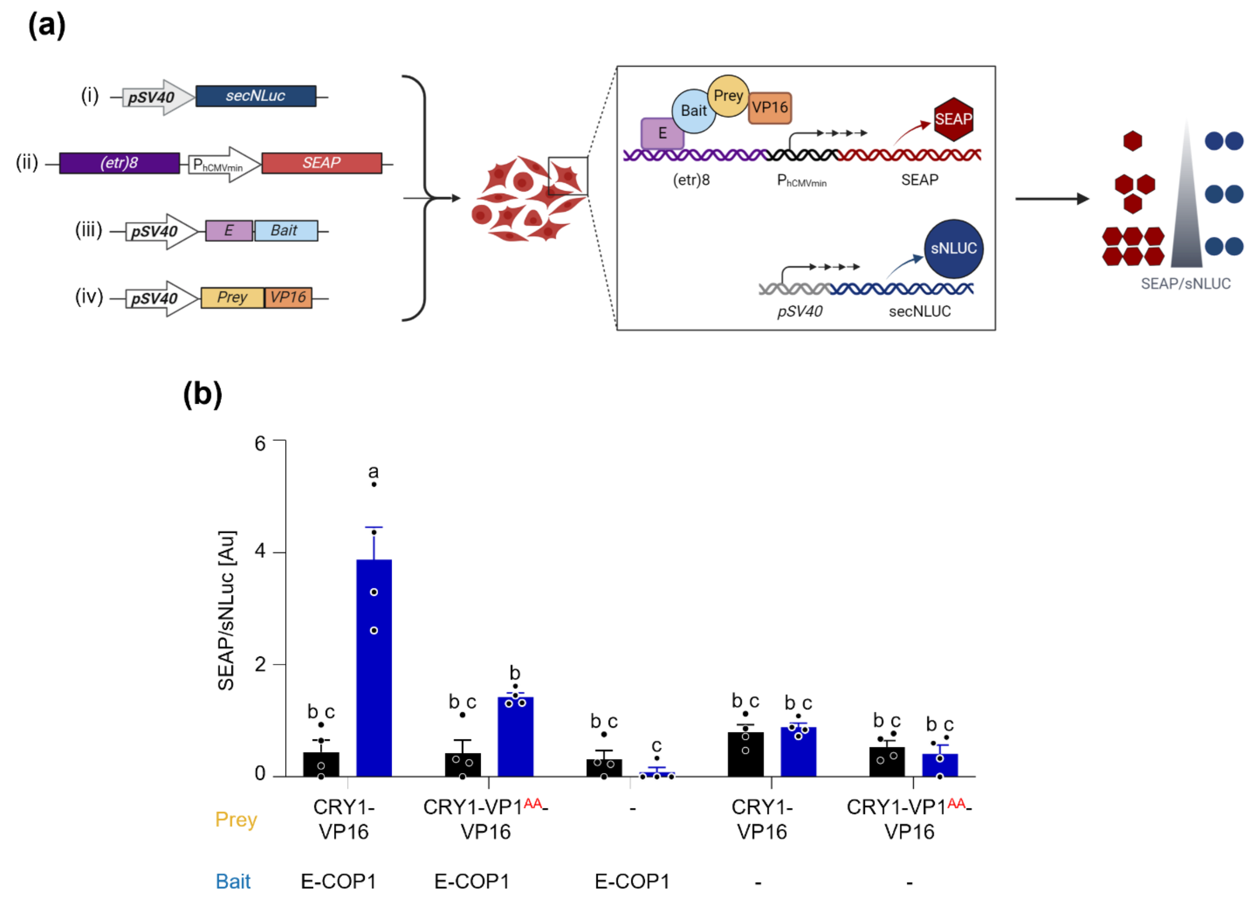

Fig. S8: CRY1 interacts with COP1 via its VP1 motif in the mammalian two-hybrid system. (a) General overview of the mammalian two-hybrid system. CHO-H1 cells were transiently co-transfected with four plasmids: (i) normalisation plasmid: expresses secreted NanoLuciferase (sNLuc) under the control of the constitutively active simian virus 40 promoter (pSV40). (ii) reporter plasmid: expresses the human placental secreted alkaline phosphatase (SEAP) under the control of minimal human cytomegalovirus promoter (PhCMVmin) fused to the octameric E-specific operator site *(etr)8.* (iii) bait plasmid: constitutively expresses the protein of interest 1 fused to the to the DNA-binding erythromycin repressor protein (E). (iv) prey plasmid: constitutively expresses the protein of interest 2 fused to the Herpes simplex virus-derived transactivation domain (VP16). Upon interaction between the bait and prey proteins, the transactivation domain VP16 is brought into close proximity of the CMV minimal promoter, recruiting the transcription machinery and activating the expression of the SEAP reporter gene. sNLuc is constitutively coexpressed for normalization. Both SEAP and sNLuc are secreted into the cell culture medium. The levels of SEAP and sNLuc are then measured and quantified as a readout for protein-protein interaction. Created in BioRender. Hoecker, U. (2024) BioRender.com/ x54q349. **(b)** CHO-K1 cells were transiently co-transfected with the SEAP reporter plasmid, the normalisation plasmid, CRY1 or CRY1-VP1^{AA} fused to VP16 and COP1 fused to E. Cells were kept in the darkness or illuminated with blue light (10 µmol m^{-2} s^{-1}) for 24 hours. SEAP and sNLuc values were determined in the cell culture medium. Dots represent the values of biological replicates. Statistical differences were estimated using or two-way ANOVA followed by Tukey's multiple comparison. Different letters indicate statistically significant differences ($p \leqslant 0.05$). Error bars indicate SEM. Dots represent the values of biological replicates. The experiment was repeated twice with similar results.

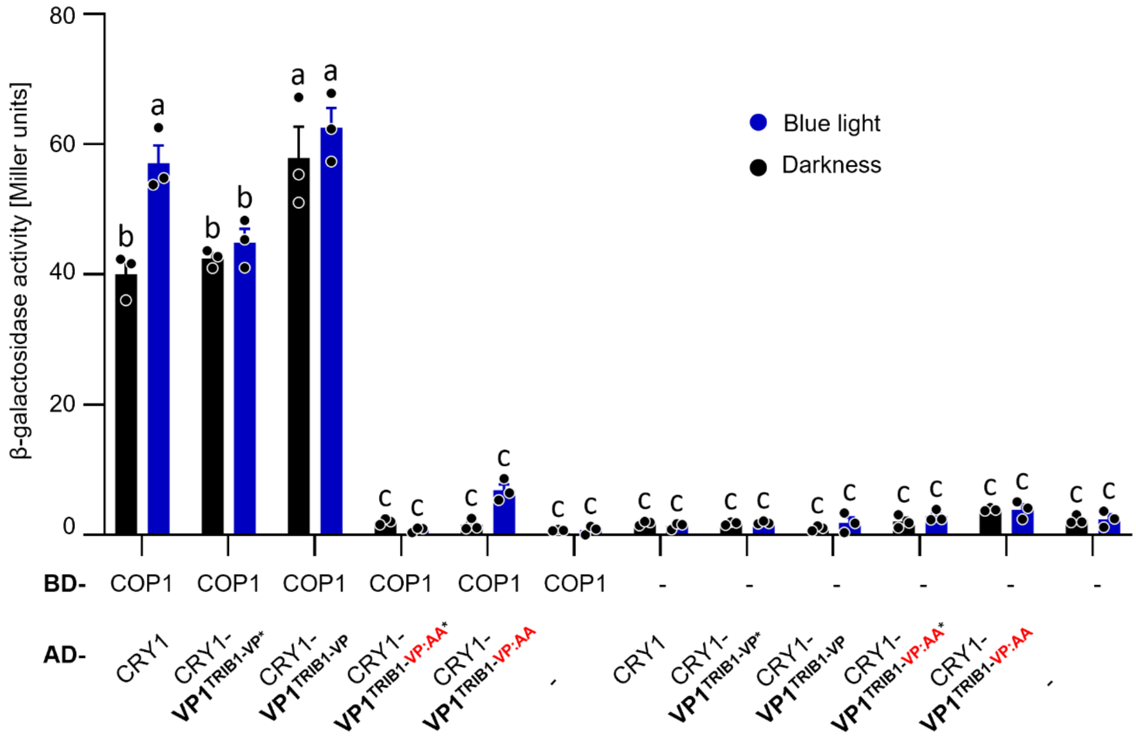

Fig. S9: CRY1-TRIB1 chimeras interact with COP1. Yeast two-hybrid assays with COP1 as bait and CRY1 variants as prey. Co-transformed yeast cells grew in darkness for 2 days (Darkness) or for 1 d in darkness, followed by exposure to 50 μmol m^{-2} s^{-1} blue light for 1 d (Blue light). Statistical differences were estimated using one-way ANOVA followed by Tukey 's multiple comparison. Different letters indicate statistical differences ($p \leqslant 0.05$). Error bars indicate SEM. Dots represent the values of biological replicates. The experiment was repeated once with similar results.

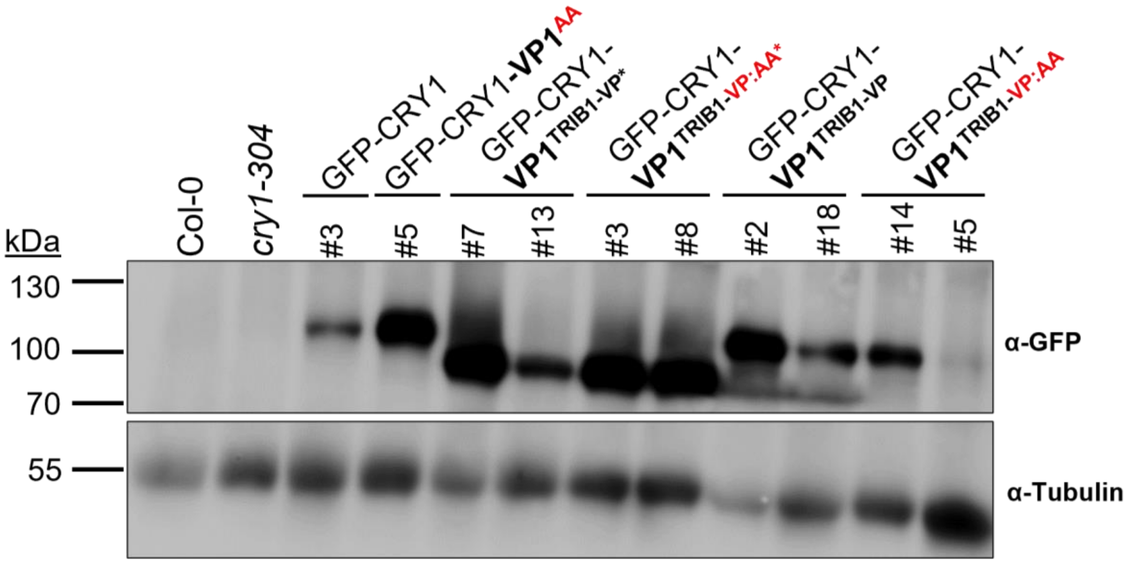

Fig. S10: Immunoblot analysis of transgenic *cry1-304* seedlings expressing GFP-CRY1, GFP-CRY1-VP1^{AA}, GFP-CRY1-VP1$^{TRIB1\text{-}VP}$*, GFPCRY1-VP1$^{TRIB1\text{-}VP:AA*,}$ GFP-CRY1-VP1$^{TRIB1\text{-}VP}$ or GFP-CRY1-VP1$^{TRIB1\text{-}VP:AA}$ under the control of the *35S* promoter. Proteins of 7-day-old seedlings grown in 2.5 µmol m^{-2} s^{-1} continuous blue light were extracted in SDS buffer. Line numbers (#) indicate independent transgenic lines. GFP-tagged proteins were detected by an α-GFP antibody. Tubulin levels detected by α-Tubulin are shown as loading control.The experiment was repeated twice with similar results

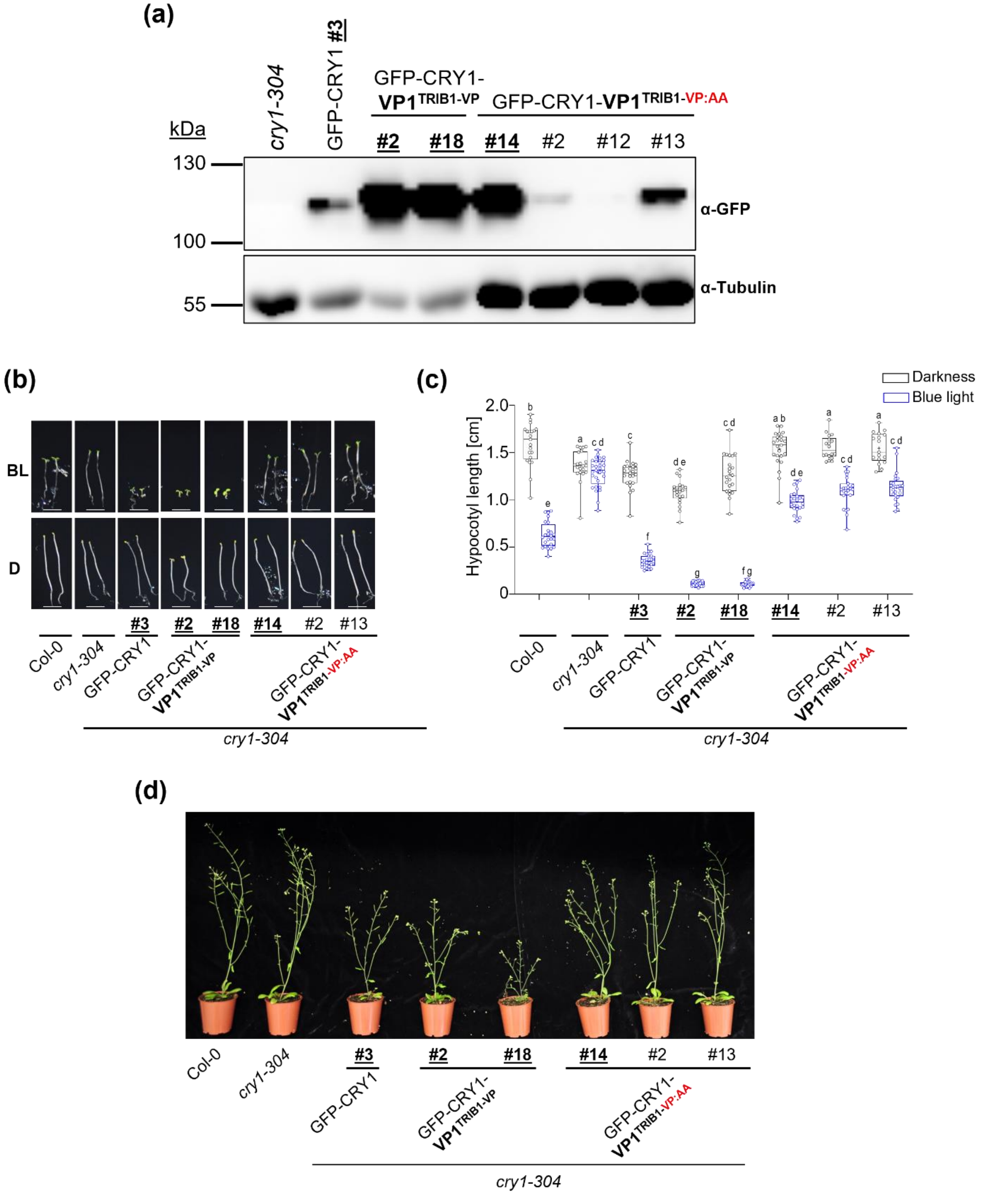

Fig. S11: None of the tested GFP-CRY1-VP1$^{TRIB1\text{-}VP:AA}$ *cry1-304* lines complemented the *cry1-304* mutant phenotype. (a) Immunoblot analysis of transgenic *cry1-304* seedlings expressing GFP-CRY1, GFP-CRY1-VP1$^{TRIB1\text{-}VP}$ or GFP-CRY1-VP1$^{TRIB1\text{-}VP:AA}$. Seedlings were grown in 2.5 μmol m^{-2} s^{-1} continuous blue light for 7 days. Line numbers (#) indicate independent transgenic lines. GFP-tagged proteins were detected by an α-GFP antibody. Tubulin levels detected by α-Tubulin are shown as loading control.

(b-d) Phenotypic analysis of transgenic *cry1-304* mutants expressing the indicated CRY1 mutant variant fused to GFP. **(b, c)** Hypocotyl length and pictures of representative 7-day-old seedlings grown in darkness (D) or 2.5 µmol m^{-2} s^{-1} blue light (BL) compared with WT (Col-0) and *cry1-304*. Box plots are shown for n > 20 seedlings. Hinges, Whiskers, median and mean are represented as explained above. Differences between genotypes were estimated using two-way ANOVA followed by Tukey's multiple comparison. Different letters indicate statistically significant differences ($p \leqslant 0.05$). (Scale bar = 0.5 cm). Data points outside the whiskers are plotted as rectangles. **(d)** Phenotype of 5-week-old adult plants grown under long-day conditions. All transgenes were expressed under the control of the 35S promoter. Transgenic lines with bold and underlined line numbers were homozygous for the transgene (T4 generations), lines with non-bold line numbers were segregating for the transgene (T3 generations).

Methods S1: Cloning of constructs used in this study

Primers used for cloning are listed in Table S1**.** All plasmids used are listed in Table S2.

The CDS of *CRY1* or *CCT1* was amplified from pDONR221-CRY1 (Holtkotte *et al.*, 2017). The CDS of *CRY1-VP1^{AA}* or *CCT1-VP1^{AA}* was amplified from pDONR221-CRY1-VP1^{AA} (Ponnu *et al.*, 2019).

1. <u>Plasmids for plant transformation</u>

The constructs for plant transformation *35S:GFP-CRY1* and *35S:GFP-CRY1-VP1^{AA}* were generated via Gateway® LR reaction using the entry clones pDONR221-CRY1 (Holtkotte *et al.*, 2017) and pDONR221-CRY1-VP1^{AA} (Ponnu *et al.*, 2019) respectively and the destination vector pFAST-R06 (Shimada *et al.*, 2010).

DNA fragments of chimeric CRY1-VP1$^{TRIB1-VP}$ constructs were generated by overhang-extension PCR. CRY1 (1-1629 bp), with a 3' overhang of the TRIB1 VP motif sequence and a 5' overhang for Gibson assembly into pENTR3C, was amplified using oLT029 and oLT032 as primers and pB42AD_CYR1 (Ponnu *et al.*, 2019) as template. Next, CRY1 (1660-2046 bp) with a 5' overhang of the TRIB1 VP motif sequence and a 3' overhang for Gibson assembly into pENTR3C was amplified using oLT031 and oLT030 as primers and pB42AD_CYR1 (Ponnu *et al.*, 2019) as template. Both fragments were then fused in an overhang extension PCR by adding both fragments in one reaction as templates and using oLT029 and oLT030 as primers. The generated fragment was cloned into BamHI and XhoI restriction sites of *pENTR3C* via Gibson assembly. To generate the chimeric CRY1-VP1$^{TRIB1-VP*}$ construct, CRY1-VP1^{TRIB1*} was amplified in an overhang PCR with pENTR3C-CRY1-VP1^{TRIB1} as template and oLT029 and oLT037 as primers. The generated

fragment was cloned into XhoI and BamHI digested *pENTR3C* via Gibson assembly. CRY1-VP1-TRIB1$^{VP:AA*}$ and CRY1-VP1$^{TRIB1-VP:AA}$ were created by introducing VP to AA mutations in the respective entry clones (pENTR3C-CRY1-VP1$^{TRIB1-VP}$ and pENTR3C- CRY1-VP1$^{TRIB1-VP*}$) trough site-directed mutagenesis using oLT130 and oLT131 (CRY1-VP1$^{TRIB1-VP:AA}$) or oLT128 and oLT129 (CRY1-VP1$^{TRIB1-VP:AA*}$) as primers. The generated entry clones pENTR3C-CRY1-VP1$^{TRIB1-VP}$, pENTR3C-CRY1-VP1$^{TRIB1-VP*}$, pENTR3C-CRY1-VP1$^{TRIB1-VP:AA}$, pENTR3C-CRY1-VP1$^{TRIB1-VP:AA*}$ were cloned into pFAST-R06 (Shimada *et al.*, 2010) via Gateway® LR reaction.

2. <u>Plasmids for co-localization and FRET-FLIM assays</u>

pAMARENA (Steffens *et al.*, 2014), pENSG-YFP and pENSG-CFP (Laubinger *et al.*, 2006) were used for N-terminal tagging with mCherry, YFP and CFP respectively. The plasmids YFP-COP1, CFP-PAP2, mCherry-PAP2, mCherry-CRY1 and mCherry-CRY1-VP1^{AA} were described before (Maier *et al.*, 2013; Ordoñez-Herrera *et al.*, 2018; Ponnu *et al.*, 2019). CFP-CRY1 and CFP-CRY1-VP1^{AA} were cloned via Gateway® LR reaction using the entry clones pDONR221-CRY1 and pDONR221-CRY1-VP1^{AA} (Holtkotte *et al.*, 2017; Ponnu *et al.*, 2019) and the destination vector pENSG-CFP. mCherry-CCT1 and mCherry-CCT1-VP1^{AA} (Ponnu *et al*, 2019) were cloned via Gateway® LR reaction using the entry clones and the destination vector pAMARENA.

3. <u>Plasmids for Luciferase-Complementation assays</u>

All cLUC and cLUC expression clones were generated by conventional digestion-ligation cloning. The *CRY1* and *CRY1-VP1^{AA}* CDS were amplified with the primer combination oLT159 and oLT160 and cloned into the pCambia1300-nLUC vector (Chen *et al.*, 2008) using KpnI and SalI restriction sites. The *PAP2* CDS was amplified with the primer combination oLT139 and oLT140 and the *HY5* CDS was amplified with the with the primer combination oLT165 and oLT164. *PAP2* and *HY5* CDS were both cloned into the pCambia1300-nLUC vector (Chen *et al.*, 2008) using KpnI and SalI restriction sites. The *COP1* CDS was amplified with primer combination oLT137 and oLT138 and cloned into the pCambia1300-cLUC vector (Chen *et al.*, 2008) using KpnI and SalI restriction sites.

To generate the GPF-NLS-GUS construct, NLS-GUS was amplified from pJP217_pFAST-R02-GUS-GW using oLT146 and oLT147 as primer pair and introduced into pENTR3C (Thermofisher) via Gibson assembly (Gibson *et al.*, 2009) using XhoI and BamHI restriction sites.

Next, the generated entry clone pENTR3C-NLS-GUS was cloned into pFAST-R06 (Shimada *et al.*, 2010), via Gateway® LR reaction (Thermofisher) to generate the GFP-NLS-GUS construct. The generation of the GFP-CRY1 and GFP-CRY1-VP1^{AA} is described in the plasmids for plant transformation section.

To generate the GFP-NLS-CCT1 construct, pENTR3C-NLS-CCT1 (Ponnu *et al.*, 2019) was cloned into into pFAST-R06 (Shimada *et al.*, 2010), via Gateway® LR reaction (Thermofisher).

To generate the GFP-NLS-GUS-CCT1 and GFP-NLS-GUS-CCT1-VP1^{AA} constructs, NLS-GUS was amplified from pJP217_pFAST-R02-GUS-GW using oLT146 and oLT235 and CCT1 or CCT1-VP1AA were PCR amplified with oLT236 and oLT030. The amplified NLS-GUS and CCT1 or CCT1-VP1^{AA} was cloned into XhoI and BamHI digested *pENTR3C* using the ClonExpress II One Step Cloning Kit (Vazyme). Next, the generated entry clones pENTR3C-NLS-GUS-CCT1 and pENTR3C-NLS-GUS-CCT1-VP1^{AA} were cloned into pFAST-R06 (Shimada *et al.*, 2010), via Gateway® LR reaction (Thermofisher) to generate the GFP-NLS-GUS-CCT1 and GFP-NLS-GUS-CCT1-VP1^{AA} constructs.

4. <u>Plasmids for yeast three-hybrid assay</u>

For Y2H studies, LexA based BD- and AD-vectors (pEG202 (pLexA)-GW and pB42ADGW) were used (Ponnu *et al.*, 2019). The clones BD-COP1 and AD-CRY1 were already described (Holtkotte *et al.*, 2017). To generate the clones AD-CRY1-VP1$^{TRIB1\text{-}VP}$, AD-CRY1-VP1$^{TRIB1\text{-}VP*}$, AD-CRY1-VP1$^{TRIB1\text{-}VP:AA}$ and AD-CRY1-VP1$^{TRIB1\text{-}VP:AA*}$, the respective entry clones generated for plant transformation were cloned into pB42ADGW (Ponnu *et al.*, 2019) via Gateway® LR reaction.

5. <u>Plasmids for yeast three-hybrid assay</u>

Y3H assays were performed in the Y190 yeast strain (Clontech™) using pBridge as bait- and pACT2-GW (Clontech) or pGADT7 as prey-vectors in GAL4 system. The yeast vector pGADT7-COP1 was generated and provided by Lau et al., (2019). To generate pACT2-AD-PAP2, the entry clone PAP2-pENTR (Maier *et al.*, 2013) was recombined into the destination vector pACT2-GW (Clontech, modified by Uhrig *et al*, (2004) using Gateway® LR reaction.

The construct pBridge-BD-COP1-E was generated by amplifying *COP1* CDS and clone it via ClonExpress II One Step Cloning Kit (Vazyme) into pBridge-E-E using EcoRI and BamHI restriction sites. To generate pBridge-BD-COP1-CRY1, pBridge-COP1-E was amplified with

oLT187 and oLT188, and *CRY1* CDS was amplified with oLT189 and oLT186. Both amplicons were used in a ClonExpress II One Step reaction to generate the final plasmid. Similary, *CRY1-VP1^AA^* was amplified with oLT189 and oLT186, *CCT1* and *CCT1-VP1^AA^* with oLT192 and oLT186 and cloned into the PCR amplified pBridge-COP1-E using the ClonExpress II One Step Cloning Kit (Vazyme). To generate the other CCT1 or CCT1-VP1^{AA} expression clones, *CCT1* or *CCT1-VP1*^{AA} were PCR amplified with oLT170 and oLT154 and ligated into Eco52I and PstI digested pBridge-E-E or pBridge-HY5-E (Lau *et al.*, 2019). The final plasmids were pBridge-E-CCT1 or pBridge-E-CCT1-VP1^{AA} and pBridge-BD-HY5-CCT1 or pBridge-BD-HY5-CCT1-VP1^{AA} respectively.

6. <u>Plasmids for mammalian two- and three-hybrid assays</u>

All plasmids for the M2H and M3H system were constructed using AQUA cloning (Beyer *et al.*, 2015). For pFK012 the insert COP1 was amplified from the template pDONR221-COP1 (Holtkotte *et al.*, 2017) using the primers oFK097 and oFK098, creating overhangs according to the backbone pJS204. The backbone was amplified using oJS178 and oJS045. Using the AQUA cloning the insert with the overhangs was inserted into the backbone.

To create the plasmid pFK0112 the insert HY5 was amplified from pDONR221_HY5 using the primers oFK104 and oFK115 and inserted into the backbone pJS207 which was amplified using the primers oJS155 and oJS093.

To generate pFK0124 first pDONR221-HY5-VP^{AA} was generated. The VP to AA mutation in pDONR221-HY5 was generated through site-directed mutagenesis and oLT198 and oLT199 as primers. Then HY5-VP^{AA} was amplified from pDONR221-HY5-VP^{AA} and inserted into the PCR amplified pJS207 as described above.

Plasmid pFK0114 was created by amplifying the insert CRY1 from pDONR221-CRY1 (Holtkotte *et al.*, 2017) using the primers oFK108 and oFK117. The insert was then inserted into the backbone pJS207 as described above. Similarly, the plasmid pFK01292 was created but amplifying the insert CRY1-VP:AA from pDONR221-CRY1-VP1^{AA} (Ponnu *et al.*, 2019).

To create plasmids pFK0129 and pFK01291 the inserts CRY1 and CRY1-VP:AA were amplified from the templates pDONR221-CRY1 (Holtkotte *et al.*, 2017) and pDONR221-CRY1-VP1^{AA} (Ponnu *et al.*, 2019) using the primers oFK130 and oFK272 and inserted into the backbone pFK0122 which was amplified using primers oJS099 and oJS045.

For the normalisation element pFK041 the insert secNLuc was amplified from the template pUU221 using the primers oFK151 and oFK152 and inserted into the backbone pLK090 which was amplified using primers oJS338 and oDD549.

Table S1: List of primers used in this study

ID	name	Sequence
**Primers to generate plasmids for plant transformation**		
ID	*name*	*Sequence*
oLT029	CRY1-gib_fwd	ggaaccaattcagtcgactgGCATGTCTGGTTCTGTATCTG
oLT030	CRY1-gib_rev	gaaagctgggtctagatatcTTACCCGGTTTGTGAAAG
oLT031	CRY1-TRIB1-ov_fwd	ttgttccagagtaccaggagTCTTTGATCAGACCTGAAGA
oLT037	CRY1-TRIB1*-gib-rev	gaaagctgggtctagatatcctctcactcctggtactctggaacaatct
oLT128	CRY1-TRIB1-VP_AA*_fwd	agatattcagatcagattGCTGCTgagtaccaggagTGAgag
oLT129	CRY1-TRIB1-VP_AA*_rev	ctcTCActcctggtactCAGCAGcaatctgatctgaatatct
oLT130	CRY1-TRIB1-VP_AA_fwd	agatattcagatcagattGCTGCTgagtaccaggagtctttg
oLT131	CRY1-TRIB1-VP_AA_rev	caaagactcctggtactCAGCAGcaatctgatctgaatatct
**Primers to generate plasmids for LCI assays**		
ID	*name*	*Sequence*
oLT030	CRY1-gib_rev	gaaagctgggtctagatatcTTACCCGGTTTGTGAAAG
oLT137	KpnI-COP1_fwd	cgcagcgGTACCatggaagagatttcgacggatcc
oLT138	SalI-Stop-COP1_rev	tgcaggtcgaCctacgcagcgagtaccagaac
oLT139	KpnI-PAP2_fwd	cgacgcgGTACCatgGAGGGTTCGTCCAAAG
oLT140	SalI-PAP2_rev	ctgcaggtcgaCATCAAGTTCAACAGTCTCTCC
oLT146	NLS-GUS_Gib_fwd	ccaattcagtcgactggatccatgcctaagaagaagagaaaggttttacgtcctgtagaa
oLT147	NLS-GUS_Gib_rev	gctgggtctagatatctcgatgtttgcctccctgctgcgg
oLT159	KpnI-CRY1_fwd	cgacgcgGTACCATGTCTGGTTCTGTATCTG
oLT160	SalI-CRY1_rev	ctgcaggtcgaCCCCGGTTTGTGAAAGCCG
oLT164	SalI-HY5_rev	tcgagcGTCGACAAGGCTTGCATCAGCATTAGAACCA
oLT165	KpnI-NLS-HY5_fwd	gctcgaGGTACCatgcctaagaagaagagaaaggttATGCAGGAACAAGCGACTAGC
oLT235	CCT1_rev	tagttgccacatctgcatttgtttgcctccctgctgc
oLT236	CCT1-GUS_fwd	cgcagcagggaggcaaacaaatgcagatgtggcaacta
**Primers to generate plasmids for Y3H assays**		
ID	*name*	*Sequence*
oLT154	PstI-CRY1_rev	tcgagcctgcagTTACCCGGTTTGTGAAAGC
oLT170	Eco52I-CCT1_fwd	gctcgacggccggtcgaatgcagatgtggcaa
oLT186	CRY1_CE II_rev	cccgaagatcctacctaggcTTACCCGGTTTGTGAAAGCC
oLT187	pB-COP1_CE II_fwd	GCCTAGGTAGGATCTTCGGGC
oLT188	pB-COP1_CE II_rev	tGCAGAGATCTACCGGCCG
oLT189	CRY1_CE II_fwd	gcggccggtagatctctgcgcATGTCTGGTTCTGTATCTGGTTGTG
oLT192	CCT1_CE_fwd	GCGGCCGGTAGATCTCTGCgcatgcagatgtggcaactagaagc

Primers to generate plasmids for M2H assays		
ID	***name***	***Sequence***
oLT198	HY5_VP-AA_fwd	GAGGAGATACGGCGAGCTGCTGAGTTTGGAGGAGAAGC
oLT199	HY5_VP-AA_rev	GCTTCTCCTCCAAACTCAGCAGCTCGCCGTATCTCCTC
oFK097	COP1_E_fwd	ctccaggcacatgcgtccgcgtacagccgcATGGAAGAGATTTCGACGGATCCGGTTGTT
oFK098	COP1_E_rev	aagcttgggctgcaggtcgactctagaCTATCACGCAGCGAGTACCAGAACTTTGATGGT
oFK104	HY5_VP16_fwd	aggtcccggatcgaattgcggccgcccaccATGCAGGAACAAGCGACTAGCTCTTTAGCT
oFK108	CRY1_VP16_fwd	aggtcccggatcgaattgcggccgcccaccatgtctggttctgtatctggttgtggttct
oFK115	HY5_VP16_rev	agacccgtaattgtttttcgtacgcgcGCgAAGGCTTGCATCAGCATTAGAACCACCACC
oFK117	CRY1_VP16_fwd	agacccgtaattgtttttcgtacgcgcGCgcccggtttgtgaaagccttctccagttcag
oFK130	CRY1_fwd	cgaattgcggccgcaggaggcgccaccatgatgtctggttctgtatctggttgtggttct
oFK272	CRY1_rev	tcgactctagattacaccttccgctttttcttgggcccggtttgtgaaagccttctccag
oFK151	sNLUC_fwd	ttttatttcaggtcccggatcggaattgcgatgaactccttctccacaagcgccttcggt
oFK152	sNLUC_rev	atgtatcttatcatgtctggatcgaagcttttacgccagaatgcgttcgcacagccgcca
oJS045	BB_pFK0122_rev	TCTAGAGTCGACCTGCAGCC
oJS093	BB_pJS207_fwd	cgcgcgcgtacgaaaaac
oJS099	BB_pFK0122_fwd	GGTGGCGCCTCCTGC
oJS155	BB_pJS207_rev	gcggccgcaattcgatc
oJS178	BB_pJS204_fwd	ggctgtacgcggacg
oJS338	BB_pLK090_fwd	gtcaattccgatccgggacc
oDD549	BB_pLK090_rev	AAGCTTCGATCCAGACATGATAAGA

Table S2: List of materials used in this study

Antibodies used in this study		
Antibody	***Source***	***Code***
α-GFP-HRP	Miltenyi Biotec (Bergisch Gladbach, Germany)	Cat: 130-091-833
α-COP1	(Balcerowicz *et al.*, 2011)	N/A
α-HSC70	Enzo Life Sciences (Lörrach, Germany)	Cat: SPA-817
α-Tubulin	Sigma-Aldrich (Munich, Germany)	Cat: T5168
α-mouse IgG-HRP	Sigma-Aldrich (Munich, Germany)	Cat: 12-349
α-rabbit IgG-HRP	ThermoFisher Scientific	Cat: 31460
**Organisms/Strains**		
Strain	***Source***	***Alias***
Plant: Col-0	Arabidopsis Biological Resource Center (ABRC)	Col-0
Plant: *cry1-304* (Col-0)	(Mockler *et al.*, 1999)	*cry1-304*
Plant: *cop1-4* (Col-0)	(McNellis *et al.*, 1994)	*cop1-4*

Plant: *35S:GFP-CRY1* (*cry1-304*)	This study	GFP-CRY1
Plant: *35S:GFP-CRY1-VP1*^{AA} (*cry1-304*)	This study	GFP-CRY1-VP1^{AA}
Plant: *35S:GFP-CRY1-VP1*^{TRIB1} (*cry1-304*)	This study	GFP-CRY1-VP1^{TRIB1}
Plant: *35S:GFP-CRY1-VP1*^{TRIB1*} (*cry1-304*)	This study	GFP-CRY1-VP1^{TRIB1*}
Plant: *35S:GFP-CRY1-VP1*$^{\text{TRIB1-VP:AA}}$ (*cry1-304*)	This study	GFP-CRY1-VP1$^{\text{TRIB1-VP:AA}}$
Plant: *35S:GFP-CRY1-VP1*$^{\text{TRIB1-VP:AA*}}$ (*cry1-304*)	This study	GFP-CRY1-VP1$^{\text{TRIB1-VP:AA*}}$
Yeast: Y190	(Harper *et al.*, 1993), Clontech	N/A
**Plasmids**		
Name	***Source***	***Alias***
pDONR221-CRY1	(Holtkotte *et al.*, 2017)	N/A
pDONR221-CRY1-VP1^{AA}	(Ponnu *et al.*, 2019)	N/A
pDONR221-COP1	(Holtkotte *et al.*, 2017)	N/A
pB42AD_CYR1	(Ponnu *et al.*, 2019)	N/A
pENTR3C-NLS-CCT1	(Ponnu *et al.*, 2019)	N/A
pENTR3C-NLS-CCT1-VP1^{AA}	(Ponnu *et al.*, 2019)	N/A
pENTR3C-CRY1-VP1^{TRIB1}	This study	N/A
pENTR3C-CRY1-VP1^{TRIB1*}	This study	N/A
pENTR3C-CRY1-VP1$^{\text{TRIB1-VP:AA}}$	This study	N/A
pENTR3C-CRY1-VP1$^{\text{TRIB1-VP:AA*}}$	This study	N/A
pENTR3C-NLS-GUS-CCT1	This study	N/A
pDONR221-HY5	This study	N/A
pDONR221-HY5-VP^{AA}	This study	N/A
pFAST-R06	(Shimada *et al.*, 2010)	N/A
pFAST-R06-CRY1	This study	GFP-CRY1 (LCI and Floral Dipping)
pFAST-R06-CRY1-VP1^{AA}	This study	GFP-CRY1-VP1^{AA} (LCI and Floral Dipping)
pFAST-R06-CRY1-VP1^{TRIB1}	This study	N/A (Floral Dipping)
pFAST-R06-CRY1-VP1^{TRIB1*}	This study	N/A (Floral Dipping)
pFAST-R06-CRY1-VP1$^{\text{TRIB1-VP:AA}}$	This study	N/A (Floral Dipping)
pFAST-R06-CRY1-VP1$^{\text{TRIB1-VP:AA*}}$	This study	N/A (Floral Dipping)
pAMARENA	(Steffens *et al.*, 2014)	mCherry (Particle Bombardment)
pENSG-YFP	(Laubinger *et al.*, 2006)	YFP (Particle Bombardment)
pENSG-CFP	(Laubinger *et al.*, 2006)	CFP (Particle Bombardment)
pENSG-YFP-COP1	(Ordoñez-Herrera *et al.*, 2018)	YFP-COP1 (Particle Bombardment)
pENSG-CFP-PAP2	(Maier *et al.*, 2013)	CFP-PAP2 (Particle Bombardment)
pAMARENA-PAP2	(Ponnu *et al.*, 2019)	mCherry-PAP2 (Particle Bombardment)
pAMARENA-CRY1	(Ponnu *et al.*, 2019)	mCherry-CRY1 (Particle Bombardment)
pAMARENA-CRY1-VP1^{AA}	(Ponnu *et al.*, 2019)	mCherry-CRY1-VP1^{AA} (Particle Bombardment)
pENSG-CFP-CRY1	This study	CFP-CRY1 (Particle Bombardment)

pENSG-CFP-CRY1-VP1^{AA}	This study	CFP-CRY1-VP1^{AA} (Particle Bombardment)
pAMARENA-CCT1	This study	mCherry-CCT1 (Particle Bombardment)
pAMARENA-CCT1-VP1^{AA}	This study	mCherry-CCT1-VP1^{AA} (Particle Bombardment)
pCambia1300-nLUC	(Chen *et al.*, 2008)	nLUC (LCI)
pCambia1300-cLUC	(Chen *et al.*, 2008)	cLUC (LCI)
pCambia1300-nLUC-CRY1	This study	nLUC-CRY1 (LCI)
pCambia1300-nLUC-CRY1-VP1^{AA}	This study	nLUC-CRY1-VP1^{AA} (LCI)
pCambia1300-nLUC-PAP2	This study	nLUC-PAP2 (LCI)
pCambia1300-nLUC-HY5	This study	nLUC-HY5 (LCI)
pCambia1300-cLUC-COP1	This study	cLUC-COP1 (LCI)
pJP217_pFAST-R02-GUS-GW	This study	N/A (Binary vector with seed RFP marker for overexpressing GUS-fusion proteins)
pENTR3C-NLS-GUS	This study	N/A
pFASTR06-NLS-GUS	This study	GFP-NLS-GUS (LCI)
pFASTR06-NLS-CCT1	This study	GFP-NLS-CCT1 (LCI)
pFASTR06-NLS-GUS-CCT1	This study	GFP-NLS-GUS-CCT1 (LCI)
pFASTR06-NLS-GUS-CCT1-VP1^{AA}	This study	GFP-NLS-GUS-CCT1-VP1^{AA} (LCI)
pEG202-(pLexA)-GW	(Ponnu *et al.*, 2019)	BD (Y2H)
pB42ADGW	(Ponnu *et al.*, 2019)	AD (Y2H)
pEG202-BD-COP1	(Holtkotte *et al.*, 2017)	BD-COP1 (Y2H)
pB42AD-CRY1	(Holtkotte *et al.*, 2017)	AD-CRY1 (Y2H)
pB42AD-CRY1-VP1$^{TRIB1\text{-}VP}$	This study	AD-CRY1-VP1$^{TRIB1\text{-}VP}$ (Y2H)
pB42AD-CRY1-VP1$^{TRIB1\text{-}VP*}$	This study	AD-CRY1-VP1$^{TRIB1\text{-}VP*}$ (Y2H)
pB42AD-CRY1-VP1$^{TRIB1\text{-}VP:AA}$	This study	AD-CRY1-VP1$^{TRIB1\text{-}VP:AA}$ (Y2H)
pB42AD-CRY1-VP1$^{TRIB1\text{-}VP:AA*}$	This study	AD-CRY1-VP1$^{TRIB1\text{-}VP:AA*}$ (Y2H)
pBridge	Clontech	N/A
pACT2-GW	(Uhrig *et al.*, 2004)	AD (Y3H)
pGADT7-COP1	(Lau *et al.*, 2019)	AD-COP1 (Y3H)
pACT2-AD-PAP2	This study	AD-PAP2 (Y2H)
pBridge-BD-COP1-E	This study	BD-COP1-- (Y3H)
pBridge-BD-COP1-CRY1	This study	BD-COP1; Bridge-CRY1 (Y3H)
pBridge-BD-COP1-CRY1-VP1^{AA}	This study	BD-COP1; Bridge-CRY1-VP1^{AA} (Y3H)
pBridge-BD-E-CCT1	This study	BD-E; Bridge-CCT1 (Y3H)
pBridge-BD-E-CCT1-VP1^{AA}	This study	BD-E; Bridge- CCT1-VP1^{AA} (Y3H)
pBridge-BD-COP1-CCT1	This study	BD-COP1, Bridge-CCT1 (Y3H)
pBridge-BD-COP1-CCT1-VP1^{AA}	This study	BD-COP1, Bridge- CCT1-VP1^{AA} (Y3H)
pBridge-BD-HY5-E	(Lau *et al.*, 2019)	BD-HY5; Bridge- (Y3H)
pBridge-BD-HY5-CCT1	This study	BD-HY5; Bridge-CCT1 (Y3H)
pBridge-BD-HY5-CCT1-VP1^{AA}	This study	BD-HY5; Bridge-CCT1-VP1^{AA} (Y3H)
pFK012_P_{sv40}-E-COP1-T_{sv40}	This study	E-COP1 (M2H)

pFK0112_ P_{sv40}-HY5-VP16-NLS-T_{sv40}	This study	HY5-VP16 (M2H)
pFK0114_P_{sv40}-CRY1-VP16-NLS-T_{sv40}	This study	CRY1-VP16 (M2H)
pFK0124_ P_{sv40}-HY5-VP^{AA}-VP16-NLS-T_{sv40}	This study	HY5-VP^{AA} (M2H)
pFK0129_ P_{sv40}-CRY1-NLS-Tsv40	This study	CRY1 (M2H)
pFK01291_P_{sv40}-CRY1-VP^{AA}-NLS-T_{sv40}	This study	CRY1-VP^{AA} (M2H)
pFK01292_ P_{sv40} -CRY1-VP^{AA}-VP16-NLS-T_{sv40}	This study	CRY1-VP^{AA}-VP16 (M2H)
pFK041_$P_{CMV(70)}$-secNLuc-T_{sv40}	This study	Normalisation element (M2H)
pKM081_etr_8-$P_{hCMVmin}$-SEAP-pA	(Müller *et al.*, 2013)	Reporter (M2H)
pWW035_P_{sv40}-E-VP16-pA	(Müller *et al.*, 2013)	Positive control (M2H)
pLH002_P_{sv40}-GFP-T_{sv40}	Lisa Hüsemann	Negative control/Stuffer (M2H)
pJS204_ P_{sv40}-E-GAI-T_{sv40}	Jonas Schön	Backbone
pJS207_ P_{sv40}-GAI-VP16-T_{sv40}	Jonas Schön	Backbone
pFK0122_ P_{sv40}-CRY1-T_{sv40}	This study	Backbone
pUU221_ P_{sv40}-sNLUC-pA	Uriel Urquiza-Garcia	Backbone
pLK090_ $P_{CMV(70)}$-PhyB-VP16-IRES-E-PIF6-T_{sv40}	Leonie Koch	Backbone

References

Balcerowicz M, Fittinghoff K, Wirthmueller L, Maier A, Fackendahl P, Fiene G, Koncz C, Hoecker U. 2011. Light exposure of Arabidopsis seedlings causes rapid de-stabilization as well as selective post-translational inactivation of the repressor of photomorphogenesis SPA2. *The Plant Journal* 65: 712–723.

Beyer HM, Gonschorek P, Samodelov SL, Meier M, Weber W, Zurbriggen MD. 2015. AQUA Cloning: A Versatile and Simple Enzyme-Free Cloning Approach (D Chatterji, Ed.). *PLOS ONE* 10: e0137652.

Chen H, Zou Y, Shang Y, Lin H, Wang Y, Cai R, Tang X, Zhou J-M. 2008. Firefly Luciferase Complementation Imaging Assay for Protein-Protein Interactions in Plants. *Plant Physiology* 146: 323–324.

Gibson DG, Young L, Chuang R-Y, Venter JC, Hutchison CA, Smith HO. 2009. Enzymatic assembly of DNA molecules up to several hundred kilobases. *Nature Methods* 6: 343–345.

Harper JW, Adami GR, Wei N, Keyomarsi K, Elledge SJ. 1993. The p21 Cdk-interacting protein Cip1 is a potent inhibitor of G1 cyclin-dependent kinases. *Cell* 75: 805–816.

Holtkotte X, Ponnu J, Ahmad M, Hoecker U. 2017. The blue light-induced interaction of cryptochrome 1 with COP1 requires SPA proteins during Arabidopsis light signaling (C Fankhauser, Ed.). *PLOS Genetics* 13: e1007044.

Lau K, Podolec R, Chappuis R, Ulm R, Hothorn M. 2019. Plant photoreceptors and their signaling components compete for COP 1 binding via VP peptide motifs. *The EMBO Journal* 38.

Laubinger S, Marchal V, Gentilhomme J, Wenkel S, Adrian J, Jang S, Kulajta C, Braun H, Coupland G, Hoecker U. 2006. *Arabidopsis* SPA proteins regulate photoperiodic flowering and interact with the floral inducer CONSTANS to regulate its stability. *Development* 133: 3213–3222.

Maier A, Schrader A, Kokkelink L, Falke C, Welter B, Iniesto E, Rubio V, Uhrig JF, Hülskamp M, Hoecker U. 2013. Light and the E3 ubiquitin ligase COP1/SPA control the protein stability of the MYB transcription factors PAP1 and PAP2 involved in anthocyanin accumulation in Arabidopsis. *The Plant Journal* 74: 638–651.

McNellis TW, von Arnim AG, Araki T, Komeda Y, Miséra S, Deng XW. 1994. Genetic and molecular analysis of an allelic series of cop1 mutants suggests functional roles for the multiple protein domains. *The Plant cell* 6: 487–500.

Mockler TC, Guo H, Yang H, Duong H, Lin C. 1999. Antagonistic actions of Arabidopsis cryptochromes and phytochrome B in the regulation of floral induction. *Development* 126: 2073–2082.

Müller K, Engesser R, Schulz S, Steinberg T, Tomakidi P, Weber CC, Ulm R, Timmer J, Zurbriggen MD, Weber W. 2013. Multi-chromatic control of mammalian gene expression and signaling. *Nucleic Acids Research* 41: e124–e124.

Ordoñez-Herrera N, Trimborn L, Menje M, Henschel M, Robers L, Kaufholdt D, Hänsch R, Adrian J, Ponnu J, Hoecker U. 2018. The Transcription Factor COL12 Is a Substrate of the COP1/SPA E3 Ligase and Regulates Flowering Time and Plant Architecture. *Plant Physiology* 176: 1327–1340.

Ponnu J, Riedel T, Penner E, Schrader A, Hoecker U. 2019. Cryptochrome 2 competes with COP1 substrates to repress COP1 ubiquitin ligase activity during *Arabidopsis* photomorphogenesis. *Proceedings of the National Academy of Sciences* 116: 27133–27141.

Shimada TL, Shimada T, Hara-Nishimura I. 2010. A rapid and non-destructive screenable marker, FAST, for identifying transformed seeds of Arabidopsis thaliana. *The Plant Journal* 61: 519–528.

Steffens A, Jaegle B, Tresch A, Hülskamp M, Jakoby M. 2014. Processing-Body Movement in Arabidopsis Depends on an Interaction between Myosins and DECAPPING PROTEIN1. *Plant Physiology* 164: 1879–1892.

Uhrig JF, Canto T, Marshall D, MacFarlane SA. 2004. Relocalization of Nuclear ALY Proteins to the Cytoplasm by the Tomato Bushy Stunt Virus P19 Pathogenicity Protein. *Plant Physiology* 135: 2411–2423.
